# A tight cold-inducible switch built by coupling thermosensitive transcriptional and proteolytic regulatory parts

**DOI:** 10.1093/nar/gkz785

**Published:** 2019-09-17

**Authors:** Yang Zheng, Fankang Meng, Zihui Zhu, Weijia Wei, Zhi Sun, Jinchun Chen, Bo Yu, Chunbo Lou, Guo-Qiang Chen

**Affiliations:** 1 MOE Key Lab of Bioinformatics, Center for Synthetic and Systems Biology, School of Life Sciences, Tsinghua University, Beijing 100084, China; 2 CAS Key Laboratory of Microbial Physiological & Metabolic Engineering and State Key Laboratory of Microbial Resources, Institute of Microbiology, Chinese Academy of Sciences, Beijing 100101, China; 3 College of Life Sciences, University of Chinese Academy of Sciences, Beijing 100149, China; 4 MOE Key Laboratory of Industrial Biocatalysis, Department of Chemical Engineering, Tsinghua University, Beijing 100084, China; 5 College of Life Science, University of Science and Technology of China, Hefei 230027, China

## Abstract

Natural organisms have evolved intricate regulatory mechanisms that sense and respond to fluctuating environmental temperatures in a heat- or cold-inducible fashion. Unlike dominant heat-inducible switches, very few cold-inducible genetic switches are available in either natural or engineered systems. Moreover, the available cold-inducible switches still have many shortcomings, including high leaky gene expression, small dynamic range (<10-fold) or broad transition temperature (>10°C). To address these problems, a high-performance cold-inducible switch that can tightly control target gene expression is highly desired. Here, we introduce a tight and fast cold-inducible switch that couples two evolved thermosensitive variants, TF_ts_ and TEV_ts_, as well as an additional *Mycoplasma florum* Lon protease (*mf*-Lon) to effectively turn-off target gene expression via transcriptional and proteolytic mechanisms. We validated the function of the switch in different culture media and various *Escherichia coli* strains and demonstrated its tightness by regulating two morphogenetic bacterial genes and expressing three heat-unstable recombinant proteins, respectively. Moreover, the additional protease module enabled the cold-inducible switch to actively remove the pre-existing proteins in slow-growing cells. This work establishes a high-performance cold-inducible system for tight and fast control of gene expression which has great potential for basic research, as well as industrial and biomedical applications.

## INTRODUCTION

Temperature is a unique input signal that is characterized by its non-invasive nature, good penetrability, low cost, and reversibility. It can be sensed by a diversity of genetic regulatory parts, including DNA, or RNA modules, transcription factors, proteases and membrane-bound proteins (1−8). Their thermosensing functions are achieved via different regulatory processes, including transcriptional initiation, translational initiation, protein and RNA degradation, ion channel activation, and so on (6,9−11). However, only two regulatory processes based on thermosensitive transcription factors and 5′ untranslated regions (5′UTR) of mRNAs have been employed to design thermoswitches for the artificial regulation of target genes (12,13). Moreover, these two processes are limited to controlling the biogenesis of RNAs and proteins rather than their degradation. Without an active degradation process to remove target proteins, a thermoswitch cannot efficiently turn off the expression of target genes or remove the pre-existing proteins under the slow- or non-growth conditions (14,15). Therefore, thermosensitive protein degradation parts, such as proteases with specific cleavage activity, are highly desirable for an advanced thermoswitch.

In general, thermoswitches can be classified into heat- and cold-inducible switches (16,17). Heat-inducible switches dominate well-studied thermosensitive regulatory systems, and are mostly based on thermolabile transcriptional repressors or heat-destabilized RNA hairpin structures within 5′UTRs of mRNA (2,12,13,18). For example, TlpA, a transcriptional repressor from *Salmonella typhimurium*, undergoes sharp, temperature-dependent uncoiling between 37 and 45°C, from a DNA binding dimer state to a free monomer state, leading to an increase of target gene transcription at high temperatures (7,19). TcI, a thermolabile mutant of bacteriophage λ repressor CI, which has been engineered to form various heat-inducible switches, binds to responsive promoters to block transcription at low temperatures and becomes inactive at high temperatures (13,20). Both transcription repressors show sharp thermal transitions, with more than 100-fold induction within 10 degrees Celsius.

However, very few cold-inducible switches have been described in either natural or engineered systems, although low temperature is good for promoting the correct folding of recombinant proteins, reducing the formation of inclusion bodies, and keeping the stability of bioactive molecules (21−25). One notable example of cold-inducible switches is the structurally rearranged 160-nucleotide-long 5′UTR of the *cspA* mRNA, which increases gene expression by stabilizing the transcript and increasing the translation initiation efficiency at low temperatures (26,27). Another classic example is the *de novo* designed short RNA thermosensor based on a 5′UTR in which an RNase E cleavage site is buried inside a hairpin at low temperatures, yet exposed to the RNase E enzyme and quickly cleaved at high temperatures (12). However, current engineered cold-inducible switches generally suffer from broad temperature transitions, narrow dynamic ranges, or severe leaky expression (13,28), which limits their wider application. Moreover, some of these switches even require small-molecule inducers, such as isopropyl-β-d-1-thiogalactopyranoside (IPTG), to improve their performance (29), and are therefore not true, pure thermoswitches.

**Figure 1.**
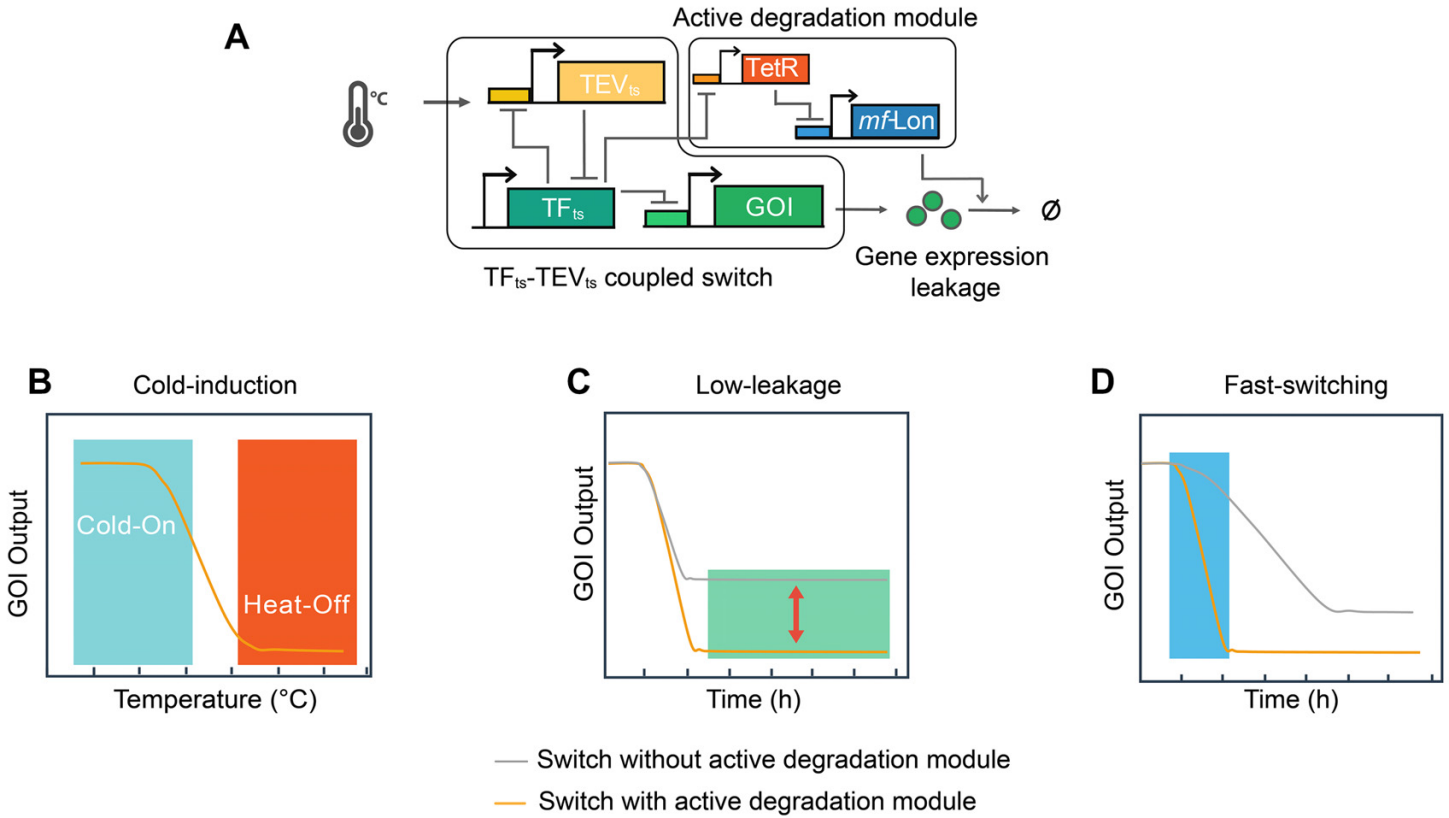
A tight cold-inducible switch composed of two thermosensitive parts. (**A**) Schematic of the high-performance cold-inducible switch that contains two modules: a basic thermoswitch and an active degradation module. The basic thermoswitch consists of mutually repressed TFts and TEVts, which regulate the expression of a gene of interest (GOI) on the transcriptional and proteolytic levels, respectively. The active degradation module includes an *mf*-Lon protease that can actively eliminate the remaining protein or that synthesized due to leaky gene expression under the control of the basic thermoswitch through TetR. Three important features of the cold-inducible switch are shown as follows: high dynamic range (**B**), low leaky expression (**C**) and fast switching (**D**).

To address these problems and develop a high-performance cold-inducible switch, we evolved two thermosensitive regulatory parts, a heat-inactivated protease and a cold-inactivated TEV-sensitive transcriptional factor, which respectively regulate gene expression at transcriptional and proteolytic levels, and combined them into a modular and tunable thermoswitch (Figure 1A). To further optimize the performance of this system, we introduced an additional proteolytic module into the switch to specifically degrade residual proteins or ones synthesized due to leaky expression (Figure 1A). The performance of the cold-inducible switch was evaluated in different bacterial species and growth media. We demonstrated the potential utility of the cold-inducible switch designed in this study by regulating the cell morphology-related genes via a temperature shift and turning on the expression of heat-unstable recombinant proteins at a low temperature to maintain their correct structure. The results showed the high-performance cold-inducible switch could tightly and rapidly regulate the target gene expression (Figures 1B–D).

## MATERIALS AND METHODS

### Bacteria and culture conditions

The *Escherichia coli* K-12 strains TOP10, MG1655, DH5α, DH10B, JM109, JM109SG and JM109SG(Δ*mreB*), the *E. coli* MG1655 strains MG1655 P_R_-MreB, MG1655 P_R_-FtsZ and MG1655 P_R_-FtsZ-pdt#4, as well as the *E. coli* B strains BL21 and Rosetta (DE3) were used in this study. Detailed information is listed in Supplementary Table S5. Genome editing was conducted using the CRISPR-Cas9 system described by Jiang *et al.* (30). The sgRNAs (single guide RNAs) and homologous recombination sequences for editing the target genes (*mreB* and *ftsZ*) are listed in Supplementary Tables S7 and S9.

Unless noted otherwise, *E. coli* strains were cultured in Luria Bertani (LB) medium with appropriate antibiotics. The antibiotics and their final concentrations used in this study were as follows: ampicillin (100 μg/ml, Inalco, Spain), chloramphenicol (25 μg/ml, Inalco), kanamycin (50 μg/ml, Inalco) and spectinomycin (100 μg/ml, Inalco), respectively. Components of culture media including LB, SOC (super optimal broth with catabolite repression), TB (terrific broth) and M9 (minimal salts), are listed in Supplementary Table S4. Strains were cultured at the required temperatures in 96-deep-well plates (1 ml medium per well) at 1000 rpm on a constant temperature microplate shaker (AllSheng, Hangzhou, China) or in 100 ml shake- flasks (20 ml medium per flask) at 200 rpm on a rotary shaker (Honor, Tianjin, China).

### Plasmid construction

All plasmids created for this study (see Supplementary Table S6) were made using Gibson Assembly (31) or Golden Gate Assembly (32), and confirmed via Sanger sequencing. Unless noted otherwise, the plasmids used in this study were built using two basic vectors as backbones, pTFA and pPA (Supplementary Figure S7), which were created in our laboratory. The vector pTFA was used to construct transcription factor-associated cassettes and pPA was used for protease-associated cassettes. The sequences for important plasmids were listed in Supplementary Table S8.

### Fluorescence measurement by flow cytometry

The induction performance and degradation dynamics of the cold-inducible switch were measured using a Calibur flow cytometer (BD Biosciences, CA, USA) with appropriate settings (FSC 440, SSC 260, FITC 480). Bacterial cells were stored in the PBS containing 2 mg/ml kanamycin to stop protein expression prior to flow cytometry analysis (33,34). At least 20 000 events were collected for each sample and the data were analyzed using FlowJo software (vX.0.7, Treestar, USA). The geometric mean of fluorescence intensity was calculated and the autofluorescence of *E. coli* cells was subtracted for each sample. All data were plotted using GraphPad Prism (Version 7.0, La Jolla, USA).

### Western blot analysis

Supernatants and precipitate of protein extracts from *E. coli* Rosetta (DE3) cells expressing the target genes were analyzed by western blotting as described previously (35). The details of protein sample preparation are shown in Supplementary Figure S5. The primary mouse anti-His and anti-GAPDH (GA1R) antibodies were purchased from TIANGEN Biotech (Beijing, China) and Abcam (UK) respectively. The secondary antibody (HRP-goat anti-mouse IgG) was purchased from Jackson (USA).

### Scanning electron microscopy (SEM)

For SEM analysis, cells from 1 ml cell of bacterial culture were harvested by centrifugation at 5000 rpm for 2 min. The pellets were then fixed with 2.5% (v/v) glutaraldehyde (Leagene, Beijing, China) overnight at 4°C. The fixed cells were washed with 0.1M phosphate buffer (pH 7.2) three times (10 min each), followed by dehydrated in ethanol solutions of 50%, 70%, 80%, 90% and three times in 100% (10 min each). Next, the pellets were treated with *tert*-butyl alcohol (Sigma, MO, USA) mixed with ethanol at a ratio of 1:1 and twice with pure tertiary *tert*-butyl alcohol (10 min each). Finally, the pellets were soaked in 15 μl tertiary tert-butyl alcohol and stored at −20°C for 10 h. The samples were lyophilized for 3 h before observations under a Quanta 200 scanning electron microscope (FEI, USA).

### High throughput screening for thermosensitive mutants

Error-prone PCR was performed on the TEV-sensitive transcription factor gene (*CI434-tevS*) on the plasmid pTFA-TF_wt_-sfGFP and the protease gene (*tev*) on pPA-TEV_wt_, respectively. The PCR products were inserted into the plasmid vectors using Gibson Assembly. The resulting *CI434-tevS* and *tev* libraries were separately used to transform *E. coli* TOP10 and *E. coli* TOP10 with the pTFA-TF_wt_-sfGFP plasmid. The strains were first cultured at the intended activation temperature (*T*_1_) overnight (∼16 h), and cells with relative high fluorescence (greater than an artificially defined threshold) were sorted using a BD Influx cell sorter (BD, USA). Subsequently, the sorted cells were cultured at the desired repressed temperature (*T*_2_) overnight, and then cells with relative low fluorescence were collected. The sorted cells were plated on LB Agar, then picked and cultured at different temperatures for further verification via flow cytometry (BD Fortessa, USA). A positive control (*E. coli* TOP10 containing the plasmid pSC101-J23119-sfGFP) and negative control (*E. coli* TOP10 containing the corresponding empty vector) were used to set the appropriate gain for the fluorescence channel.

## RESULTS

### Directed evolution of the thermosensitive transcription factors and proteases

Since the well-studied cold-inducible switches are mostly based on RNAs, we sought to explore different regulatory levels, such as the transcriptional level and the proteolytic level, to expand the cold-sensing toolbox and design the thermosensitive switch depicted in Figure 1A. We selected the protease from tobacco etch virus (TEV) and the CI repressor from bacteriophage 434 (CI434) as our proof-of-concept system since they are well-studied, successfully expressed and widely used in many organisms (36−40). TEV is a highly sequence-specific cysteine protease that cleaves the amino-acid sequence ENLYFQG/S between Q and the last amino acid (G/S) (41). The natural TEV protease readily cleaves itself to generate a truncated enzyme with greatly diminished activity (42), while the TEV_S219V_ mutant is not only far more stable than the natural TEV protease, but also a more efficient catalyst (42). Thus, we used the TEV_S219V_ mutant as the parent enzyme (named TEV_wt_ in this study) for directed evolution. CI434 consists of an N-terminal DNA binding domain (R1–69), a C-terminal dimerization domain (R96–209), and a flexible linker region between them (43,44), which regulates transcription via acting on a P_R_ and P_L_ operator–promoter. In order to construct a TEV-sensitive transcription factor, we inserted the TEV cleavage site (ENLYFQG) into the linker region of CI434 between R69 and R70, resulting in a TEV-sensitive CI434 variant, CI434-tevS, which was used as the parent transcription factor in this study and referred to as TF_wt_ when compared with other derived mutants (Supplementary Figure S1). It was confirmed that CI434-tevS was specifically inactivated by TEV-mediated proteolytic degradation rather than the temperature shift (Supplementary Figures S2 and S3).

**Figure 2.**
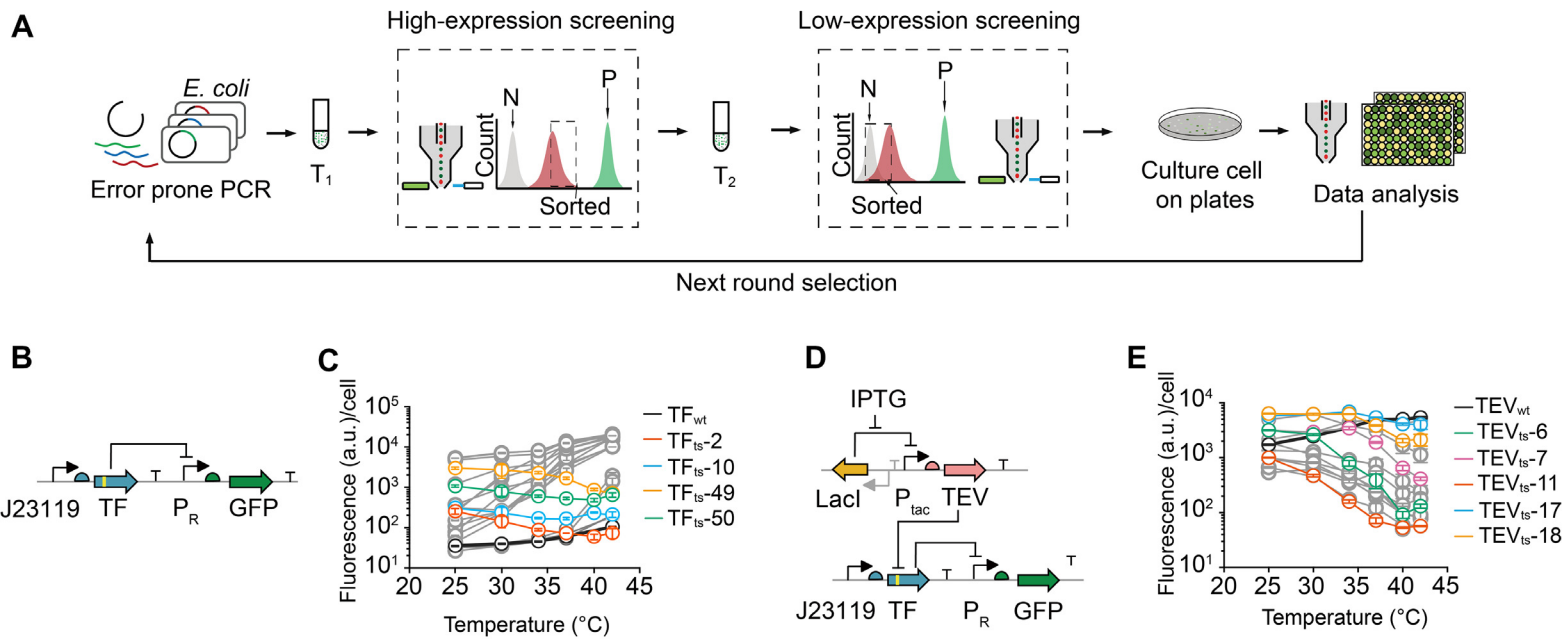
Directed evolution of the thermosensitive transcription factors and TEV proteases. (**A**) Schematic of the experimental procedures for error-prone PCR followed by positive and negative selection of desired thermosensitive mutants (see Materials and Methods for details). P: positive control; N: negative control; *T*_1_: activation temperature; *T*_2_: repression temperature. (**B**) The designed circuit for screening thermosensitive transcription factor mutants, TFts. (**C**) Fluorescence of the wild-type and selected TF mutants as a function of temperature. Gray curves represent the mutants that failed to meet the screening requirements. (**D**) The circuit for screening thermosensitive TEV protease mutants, TEVts. (**E**) Fluorescence of the wild-type and selected TEV mutants as a function of temperature. Grey curves represent the other mutants (colored curves). All the experiments were repeated at least three times, and error bars represent the SEM. a.u. arbitrary units. J23119 is a constitutive promoter. The numbers behind TFts and TEVts indicate the numbered mutants.

We developed a high-throughput screening method to select desired thermosensitive mutants based on fluorescence (Figure 2A). We first built two circuits for screening the desired mutants based on cell GFP expression (Figures 2B and D). The thermosensitive CI434-tevS transcription factor (TF_ts_) mutants were screened based on the principle that an active TF_ts_ could inhibit the expression of the reporter gene, superfolder green fluorescent protein (*sfgfp)*. Therefore, the weaker the fluorescence the higher the activity of the TF_ts_, and vice versa (Figure 2B). The thermosensitive TEV (TEV_ts_) mutants were screened based on the principle that an active TEV_ts_ could cleave the TF_wt_ to promote *sfgfp* expression. Thus, the stronger the fluorescence the higher the activity of the TEV_ts_ (Figures 2D). In order to combine TF_ts_ and TEV_ts_ into a cold-inducible switch, the desired TF_ts_ should be cold-inactivated, while the desired TEV_ts_ should be heat-inactivated, which would both reduce the fluorescence of the cells at high temperatures.

When evolving thermosensitive transcription factors, it was found that heat-inactivated mutants were quite easily enriched, but only four cold-inactivated TF_ts_ mutants were identified in five screening cycles (Figure 2C). These cold-inactivated TF_ts_ mutants showed 2- to 4-fold induction across temperatures ranging from 25 to 42°C (Supplementary Table S2). By contrast, a large number of heat-inactivated TEV_ts_ mutants were acquired, which could reduce the expression of the reporter gene more than 10-fold after shifting the temperature from 25 to 42°C (Figure 2E). From these variants, several mutants with different dynamic ranges or temperature-transition points were selected, including four transcription factors (TF_ts_-2, TF_ts_-10, TF_ts_-49 and TF_ts_-50) and five proteases (TEV_ts_-6, TEV_ts_-7, TEV_ts_-11, TEV_ts_-17 and TEV_ts_-18) (Figures 2C and E, Supplementary Table S2). The temperature-transition point was defined as the temperature at which the fluorescence intensity is reduced to 20% of the maximum. The amino acid substitutions identified in these mutants are listed in Supplementary Table S1.

### Construction of a high-performance cold-inducible thermoswitch

**Figure 3.**
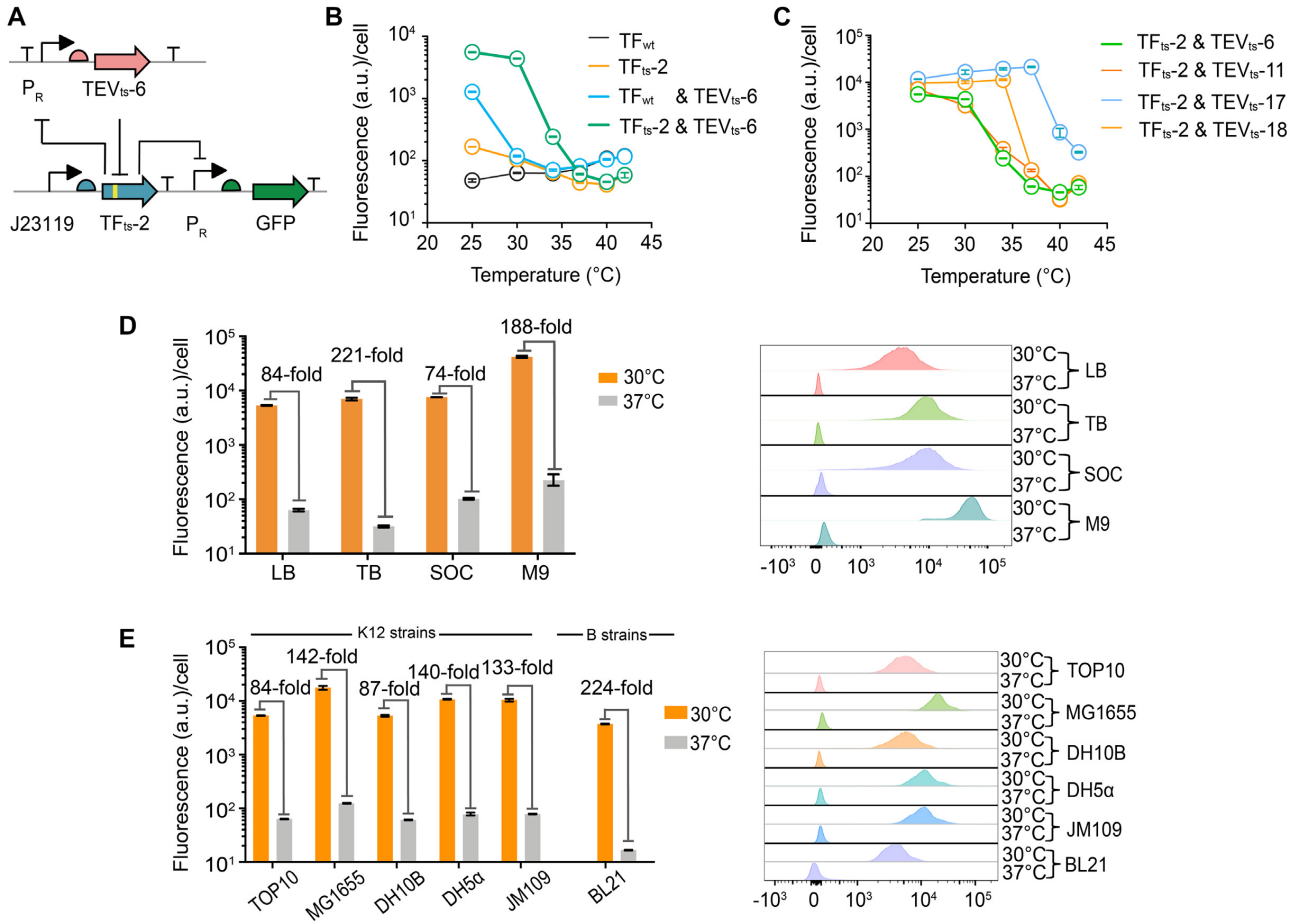
High performance of the combined cold-inducible switch. (**A**) The genetic circuit of the cold-inducible switch incorporating the new evolved TFts-2 for control at the transcriptional level and TEVts-6 at the proteolytic level. (**B**) The induction curves of the designed cold-inducible switch and its controls: TFwt is the wild-type CI434 repressor with an inserted TEV cleavage site; TFts-2 is a selected cold-inactivated CI434 repressor; TFwt&TEVts-6 is the combination of the thermosensitive TEVts-6 mutant and the non-thermosensitive TFwt; TFts-2&TEVts-6 contains thermosensitive mutants of both the TF and the protease. (**C**) The induction curves of the combined cold-inducible switches with the same TFts-2 and different TEVts. Different combinations showed diverse transition temperatures. (**D**) Quantitative measurement of the switches at 30 and 37°C in four different culture media (LB, TB, SOC and M9). (**E**) Quantitative measurement of the switches at 30 and 37°C in six different *E. coli* strains in Luria Bertani (LB) medium. All the experiments were repeated at least three times, and error bars represent SEM. a.u. arbitrary units.

Using the evolved thermosensitive transcription factors and proteases, we designed the TF_ts_-TEV_ts_ coupled cold-inducible switch shown in Figure 1A, which was expected to display a multiplicative effect of the transcriptional and proteolytic thermosensors. In the switch, TF_ts_ and TEV_ts_ were mutually suppressed. The expression of TEV_ts_ and the reporter gene *sfgfp* is under the control of the TF_ts_-repressed P_R_ promoter, while the constitutively-expressed TF_ts_ is cleaved and inactivated by TEV_ts_. At low temperatures, the cold-inactivated TF_ts_ cannot tightly repress the expression of *sf*GFP and TEV_ts_, and thus TEV_ts_ can cleave the TF_ts_ to further promote the expression of *sf*GFP. At high temperatures, heat-inactivated TEV_ts_ cannot cleave TF_ts_ so the constitutively-expressed TF_ts_ turns off the transcription of TEV_ts_ and *sf*GFP. The thermosensitive mutants TEV_ts_-6 and TF_ts_-2 were first chosen to build the cold-inducible switch (Figure 3A). This switch exhibited approximately 120-fold induction when the temperature was shifted from 40 to 25°C (Figure 3B and Supplementary Table S3). By contrast, the control circuits containing only one of the thermosensors yielded only 4- or 34-fold induction with the same temperature shift (Figure 3B). Notably, the 120-fold dynamic range of the cold-inducible switch was close to the multiplication of the induction ranges of the two control circuits (4*34 = 136-fold), indicating that the combination of the two thermosensors had a linear multiplicative effect in our designed circuit.

Synthetic biology applications may have a variety of thermal requirements. It is thus highly desirable to be able to tune switches to function at different temperatures. To investigate the modularity of our system, we replaced the TEV_ts_-6 mutant with other mutants to tune the temperature-transition point of the combined switches. In this way, a series of thermosensitive switches with different transition temperatures were obtained (Figure 3C). Their corresponding temperature-transition points were 32.5°C (TEV_ts_-11 & TF_ts_-2), 33.5°C (TEV_ts_-6 & TF_ts_-2), 36.5°C (TEV_ts_-18 & TF_ts_-2), and 39.5°C (TEV_ts_-17 & TF_ts_-2), and the 10–90% transition ranges (T_10–90_), defined as the temperature difference of 90% fluorescence intensity and 10% fluorescence intensity, were 7.5, 4, 4, and 3°C, respectively. The defined T_10–90_ index indicated that these switches had sharp thermal transitions, and ∼100-fold induction was achieved within less than ten degrees (Figure 3C).

It is important to maintain the designed function of synthetic circuits and standard parts in different environments and host contexts. To evaluate the robustness of the cold-inducible switch, we measured the performance of the cold-inducible switch containing TEV_ts_-6 and TF_ts_-2 in different growth media and different *E. coli* strains. The performance of the switch was evaluated by the relative induction of the reporter gene expression between 30 and 37°C. Firstly, the induction performance of the cold-inducible switch was tested in different culture media including three nutrient-rich media (LB, TB and SOC) and one defined medium (M9). The results showed that the switch achieved excellent induction in all tested growth media, reaching 84-fold in LB medium, 221-fold in TB medium, 74-fold in SOC medium and 188-fold in M9 medium (Figure 3D). Subsequently, we tested the induction performance of the switch in several different *E. coli* strains in LB medium, including four *E. coli* K12-strains (DH10B, MG1655, DH5α, JM109) and one *E. coli* B strain (BL21). Two strains (DH10B and MG1655) were found to have similar or larger relative induction values (Figure 3E), but the other three strains DH5α, JM109 and BL21 showed little or no induction effect (Supplementary Figure S4). It is possible that the various genetic backgrounds, differences of cellular metabolism and physiology among the *E. coli* strains (45), influenced the gene expression ratios of TF_ts_-2 and TEV_ts_-6, and subsequently affected the performance of the thermoswitch. For example, if TEV_ts_-6 was expressed too much or TF_ts_-2 was expresses too little, the switch might remain in the ‘ON’ state, and the reporter gene would keep being expressed at different temperatures. To regain the function of the thermoswitch, the relative expression levels of the two regulatory proteins should be tuned. However, tuning the expression of TF_ts_-2 may influence its repression performance on the P_R_ promoter, leading to unexpected leakage of the switch. Therefore, we focused on fine-tuning the expression of TEV_ts_-6 by varying the strength of its ribosome binding site (RBS) using the RBS-Calculator software (46,47). As expected, after fine-tuning the RBS strength of TEV_ts_-6 protease (Supplementary Table S7), all the failed cases regained >100-fold induction (Figure 3E). These results indicated that the switch can achieve the desired function in different culture media and *E. coli* strains with simple RBS tuning.

### The cold-inducible switch efficiently regulated the expression of recombinant proteins and bacterial morphology

One key feature of the switch is its ability to highly express the target gene at a low temperature, which is suitable for the production of heat-unstable proteins since low temperatures can increase the stability and solubility of recombinant proteins (48). We chose three important human proteins, peptidyl-prolyl *cis*-trans isomerase G (PPIG), human spliceosomal DEAD-box protein (Prp28) and basic human fibroblast growth factor (bFGF) (Supplementary Table S10), that were reported to form insoluble and inactive inclusion bodies in *E. coli* when expressed at temperatures above 30°C. We replaced the reporter gene in the optimized cold-inducible switch for the BL21 strain with the three respective human genes and transformed the BL21 derivative strain *Rosetta* (DE3) with the resulting plasmids to express the corresponding proteins. The *Rosetta* strain compensates the tRNAs for six rare codons commonly used by eukaryotes but rarely used by *E. coli* (49) (Supplementary Figure S5A). Protein expression levels were compared by western blotting using the housekeeping protein glyceraldehyde-3-phosphate dehydrogenase (GAPDH) as loading control. The result showed that PPIG_1–175_ protein was highly expressed at 30°C but undetectable at 37°C (Supplementary Figures S5B and C). Similarly, the other two recombinant proteins were also exclusively expressed at the low temperature but not at the high temperature (Supplementary Figures S5B and C).

**Figure 4.**
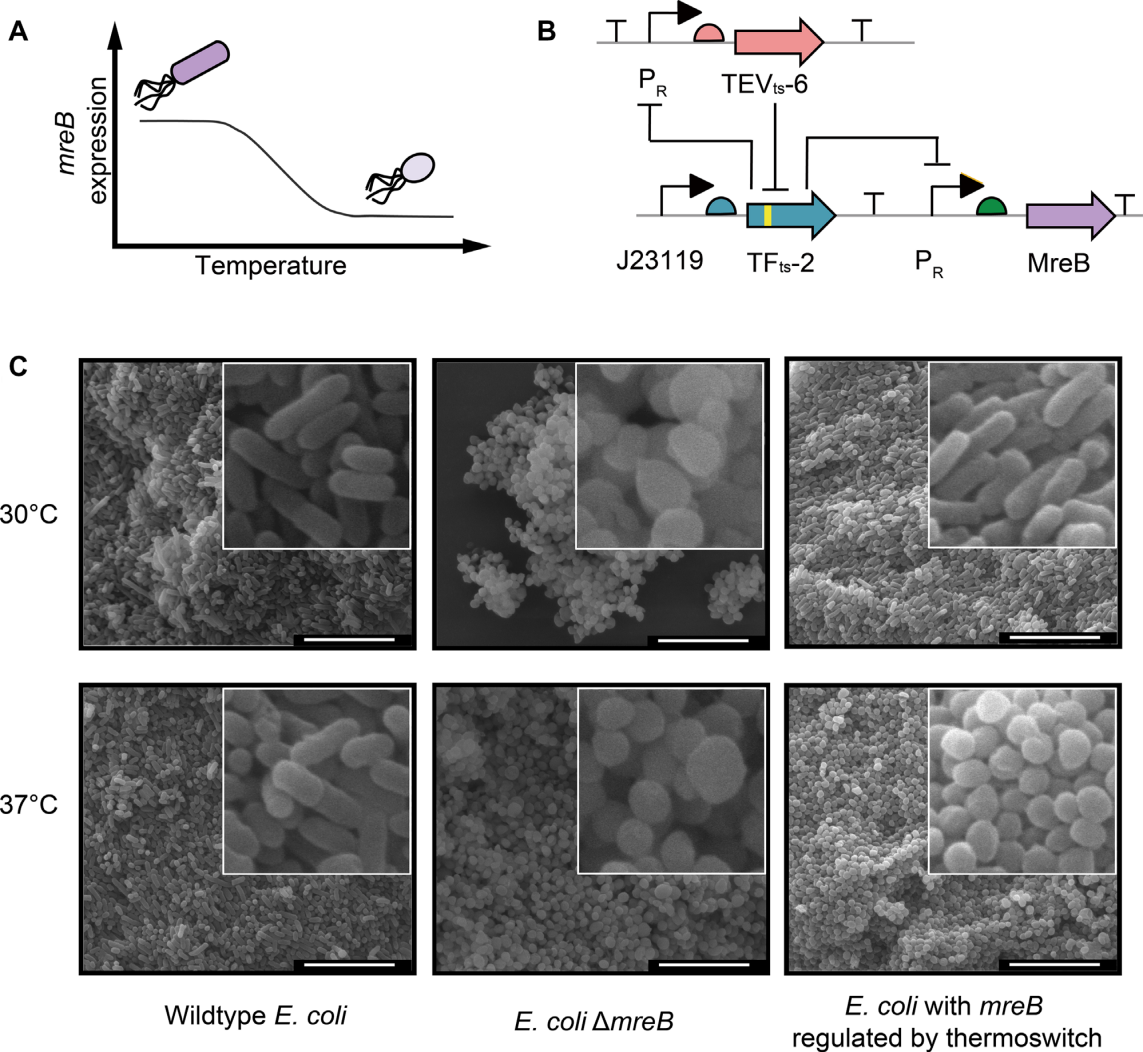
Conditional knockdown of a morphogenetic gene (*mreB*) using the cold-inducible switch. (**A**) Diagram illustrating the knockdown of the mreB gene regulated by the cold-inducible switch as the function of temperature. (**B**) The genetic circuit of the cold-inducible switch used to control the mreB gene. (**C**) The SEM images of the cell morphology of the *E. coli* strain with the cold-induced *mreB* gene, as well as its positive and negative controls at two different temperatures. Scale bar, 10 μm.

**Figure 5.**
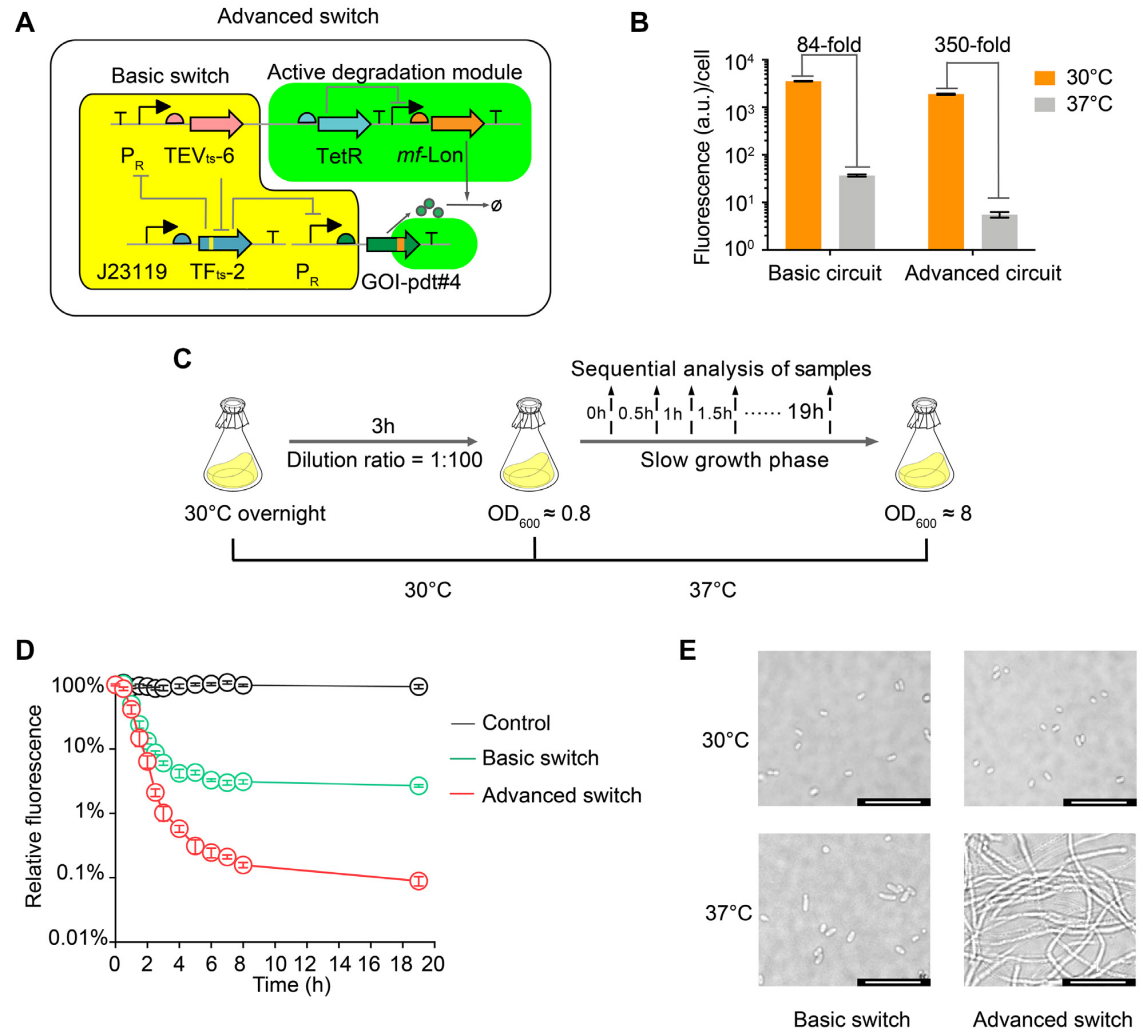
An additional active degradation module eliminates leaky gene expression and enables a fast switching process from high to low protein levels in slow growing cells. (**A**) Circuit diagram of the advanced cold-inducible switch. (**B**) Expression of reporter gene at 30 and 37°C under the control of the basic and advanced cold-inducible switches. (**C**) Experimental workflow of temperature shifting from 30 to 37°C to measure the expression of the reporter gene under the control of the advanced switch in the slow-growth phase. Samples were taken at 0, 0.5, 1, 1.5, 2, 2.5, 3, 4 and 19 h after the temperature shift. (**D**) Degradation dynamics of the basic and the advanced switches after shifting the temperature from 30 to 37°C. For comparison of the degradation dynamics of the two circuits, fluorescence (arbitrary units) of each sample at 0 h was set to 100%. (**E**) Images of the cell morphology in strains in which FtsZ expression is regulated by the basic and the advanced switch at 30 and 37°C. Scale bar, 10 μm. All the experiments were repeated at least three times, and the error bars represent SEM.

In addition to the highly efficient expression of recombinant proteins, the cold-inducible switch could also be used as a tool to noninvasively and efficiently regulate endogenous bacterial genes. We first targeted a conditionally essential morphogenetic gene, *mreB*, which codes for a cytoskeletal protein required for maintaining the rod shape of bacilli. Deletion or weak expression of *mreB* results in round cells (50,51), which increases the bacterial cell volume and could subsequently enhances the accumulation of intracellular products, such as the biodegradable polyester polyhydroxybutyrate (PHB) (52,53). To design the cold-sensitive *mreB* expression cassette, the reporter gene of the TF_ts_-2 & TEV_ts_-6 cold-inducible switch was replaced with the *mreB* gene, and the corresponding construct was used to transform the *E. coli* strain JM109SGΔ*mreB* (Figure 4A), a *mreB*-deleted strain from our previous work (52). The results showed that our cold-inducible switch successfully converted the rod-shaped bacteria into spherical ones as the temperature changed from 30 to 37°C. By contrast, the wild-type strain, *E. coli* JM109SG, maintained a rod-like morphology at both 30 and 37°C, and the *mreB* knockout strain, *E. coli* JM109SGΔ*mreB*, maintained the same round shape at any measured temperature (Figure 4B). In this case, the *mreB* gene was deleted from chromosome and then regulated and expressed on a plasmid. However, for other essential genes that cannot be deleted from the chromosome, this operation is impractical. In order to prove the feasibility of regulating genes in situ on the chromosome, we exchanged the *mreB* promoter on the chromosome for the P_R_ promoter of the thermoswitch using the CRISPR–Cas9 system (30) and constructed a new strain, MG1655 P_R_-MreB (Supplementary Figure S6A and Supplementary Table S9). The results showed that the switch could effectively convert the morphology of the MG1655 P_R_-MreB strain from rod to spherical when the temperature was shifted from low to high, indicating this switch can also be used to control chromosomal genes (Supplementary Figure S6B). Next, we targeted another morphology-related essential gene, *ftsZ*. The FtsZ protein forms a ring-like structure (Z-ring) which is essential for cell division (54). Cells with inhibited *ftsZ* expression fail to assemble the Z ring and become long undivided filaments, resulting in slow cell growth (55,56). We employed the same TF_ts_-2 & TEV_ts_-6 cold-inducible switch to regulate the *ftsZ* gene in situ in the engineered strain MG1655 P_R_-FtsZ. However, we found that the shape of the bacteria became elongated just for a short while (data not shown), but the shape reverted soon afterwards (Figure 5E). This observation indicated that in slow-growth bacteria the leaky expression of FtsZ was enough for it to reach the critical concentration required for assembling the Z-ring to divide cells. We speculated that the newly synthesized or pre-existing target proteins could not be quickly removed after switching off the expression under slow-growth conditions due to the weak dilution in slowly dividing cells. We next tried to solve the switching-off problem of the remaining proteins, especially in slow-growing cells.

### An additional proteolytic module enabled the cold-inducible switch to tightly and quickly repress essential genes in slow-growing cells

To solve the problem of leaky expression and at the same time remove the pre-existing proteins, we introduced an additional proteolytic module into the cold-inducible switch to specifically degrade the pre-existing target proteins or those synthesized due to leaky expression, resulting in an advanced cold-inducible switch (Figure 5A). To distinguish these two versions of the cold-inducible switches, we named the former the basic switch, and the latter the advanced switch (Figure 5A). The additional proteolytic module was composed of an *mf-*Lon protease (*Mycoplasma florum* Lon protease) and a TetR repressor. The *mf-*Lon protease is orthogonal to endogenous *E. coli* proteases and can specifically degrade proteins fused to a specific 27-amino acid tag (pdt#4): AANKNEENTNEVPTFMLNAGQANHAQP (57,58). TetR was used as a signal inverter to maintain antiphase expression of the target protein and the *mf-*Lon protease. Therefore, at low temperature, the P_R_ promoter highly expresses the target gene and can drive the expression of the *tetR* gene to inhibit the expression of the *mf*-lon protease gene. Conversely, at high temperature the expressions of *tetR* and the target gene is inhibited, and the P*tet* promoter can highly express the *mf*-Lon protease to further degrade the target protein fused with the pdt#4 tag (Figure 5A). We expected that the active proteolytic capability of the additional module would not only reduce the leaky expression of target genes in the steady state, but also remove the remaining proteins under slow or non-growth conditions.

To evaluate the induction performance and leaky expression of the advanced switch, we firstly engineered the advanced switch to regulate the green fluorescence protein GFPmut3, which was fused with the optimized *mf*-Lon degradation tag *pdt#4* (57). Compared with the basic switch, the dynamic range of the advanced switch increased 4-fold, and the minimal expression at 37°C decreased >10-fold (Figure 5B). We also evaluated the active degradation of the target gene by the advanced switch in slow-growing cells, which was very important for switchable fermentation processes (59,60). In the experiment, the cell culture was allowed to gradually grow from an OD_600_ of 0.8–8 in a flask with 20 ml LB medium. After shifting the temperature from 30 to 37°C, the expression of the reporter protein was measured by flow cytometry at different time points (Figure 5C). Within the first hour, the degradation rate of the basic and the advanced switch was similar. However, the degradation rate of the basic switch subsequently decreased, while the advanced switch went on maintaining fast degradation for the following hours. As a result, the repression ability of the basic switch in the slow-growing cells was only 20-fold, while that of the advanced switch was close to 1000-fold, demonstrating the advanced switch could efficiently and quickly degrade the pre-existing protein and minimize leaky expression in slow-growing cells (Figure 5D).

We next utilized the advanced switch to control the essential morphology-related gene *ftsZ*. At first, the native promoter of the chromosomal *ftsZ* gene was replaced with the P_R_ promoter, and the *pdt#4* degradation tag was added to its coding sequence (Supplementary Table S7), resulting in a new strain, MG1655 P_R_-FtsZ-pdt#4. After introducing the advanced switch into the engineered MG1655 P_R_-FtsZ-pdt#4 strain, the normal rod-shaped bacterial cells became very long filaments after shifting the temperature from 30 to 37°C, and remained filamentous even after 24 h, indicating that the cell division of the bacterium was efficiently inhibited (Figure 5E). By contrast, after introducing the basic switch into the engineered MG1655 P_R_-FtsZ-pdt#4 strain, the bacteria still kept their rod shape at both 30 and 37°C even after 24 h. This result demonstrated that the advanced switch could effectively decrease the leaky expression of *ftsZ* and worked well in slow-growing cells.

## DISCUSSION

As temperature is a low-cost, non-toxic and easy-to-manipulate environmental signal with good penetration, thermosensitive genetic switches have been considered to hold great potential in many applications, such as cell-based therapy and industrial fermentation (13,61,62). Unlike the extensively studied heat-inducible switches, few cold-inducible switches are available in the toolbox of synthetic biology. To address this problem, a novel high-performance cold-inducible switch was built from scratch in this study. We first evolved two new thermosensitive regulatory parts. One of them is a heat-inactivated protease, and the other is a cold-inactivated transcription factor, both of which are compatible with the cold-induced 5′UTR of mRNA functioning as a switch on the translational level. Therefore, further cold-inducible switches could integrate the benefits of all three thermosensitive regulatory parts on the transcriptional, translational and proteolytic levels. In addition, the same strategy can also be applied to other commonly used transcription factors (TetR, LacI, LuxR) and proteases, such as the *Potyviridae* proteases TVMV and SuMMV (63), or human rhinovirus 3C protease R3C (64). Those potential thermoswitches are expected to be orthogonal to each other and could function in a wide range of organisms. We envision that the thermoswitches designed here will expand the thermosensitive regulator toolbox in synthetic biology because they can easily be incorporated into more sophisticated regulatory networks in both natural and synthetic systems.

We demonstrated that the switch could work in different media and different *E. coli* strains. Moreover, it could also work in other bacterial species, such as a promising industrial strain, *Halomonas bluephagenesis* TD01 (65), after fine-tuning the promoter and RBS to match the expression of the two thermosensitive parts (data not shown). Additionally, the two critical proteins used in the switch, TEV protease and CI434 repressor, are functional in both prokaryotic and eukaryotic cells (39,66−70). We therefore speculate that the cold-inducible switch would be functional in many different cell lines, even in mammalian cells. It is quite possible to develop a next-generation cold-inducible system for the Chinese Hamster Ovary (CHO) cell line, which is the workhorse for 70% of today's industrial production of therapeutic proteins (71,72).

One unique feature of our thermosensitive switch is the incorporation of proteolytic modules to improve the performance of a transcriptional switch. Commonly, a critical challenge for engineered thermosensitive switch is to improve the dynamic range of induction. There are many ways to improve the dynamic range of a genetic switch, including positive feedback loops, recombinases (73−75), layered multiple NOT gates (76), phosphorylation (77,78), non-coding RNAs (79), incorporation of decoy operators (78,80), and upstream activating sequences (81). The incorporation of two proteolytic modules into transcriptional switches introduces new levers of regulation that in principle could improve the performance of many types of dynamic circuits.

Another striking advantage of the protease-based thermoswitch is the ability to effectively switch off the target genes in slow-growing cells due to its regulation on the degradation level rather than the re-synthesis level. This feature is very important for large-scale fermentations, in which the switching usually occurs at the end of the growth phase, during which most cultured cells enter a slow-growing stage. Additionally, low temperature increases oxygen solubility in the culture medium and reduces the cellular oxygen demand, which enables the cells to grow to a higher density (21,82). Therefore, we believe that these cold-inducible switches have great potential in industrial and other application fields, especially for the production of therapeutic proteins, thermolabile chemicals and bioactive natural products.

## Supplementary Material

gkz785_Supplemental_File
