# Supplementary material for: A tight cold-inducible switch built by coupling thermosensitive transcriptional and proteolytic regulatory parts
Source: Nucleic Acids Res. 2019 Sep 17;47(21):e137. doi: 10.1093/nar/gkz785 (PMC6868347; doi:10.1093/nar/gkz785)
Supplement: gkz785_Supplemental_File [file gkz785_supplemental_file.pdf]

A tight cold-inducible switch built by coupling thermosensitive transcriptional and proteolytic regulatory parts

Yang Zheng[1†], Fankang Meng[2,3†], Zihui Zhu[1], Weijia Wei[2,3], Zhi Sun[2,3], Jinchun Chen[1,5], Bo Yu[2*], Chunbo Lou[2,3,4*], Guo-Qiang Chen[1,5*]

[1]MOE Key Lab of Bioinformatics, Center for Synthetic and Systems Biology, School of Life Sciences, Tsinghua University, Beijing 100084, China

[2]CAS Key Laboratory of Microbial Physiological & Metabolic Engineering and State Key Laboratory of Microbial Resources, Institute of Microbiology, Chinese Academy of Sciences, Beijing, 100101, China

[3]College of Life Sciences, University of Chinese Academy of Sciences, Beijing, 100149, China

[4]College of Life Science, University of Science and Technology of China, Hefei 230027, China

[5]MOE Key Lab of Industrial Biocatalysis, Dept of Chemical Engineering, Tsinghua University, Beijing 100084, China

* To whom correspondence should be addressed. Tel: +86 010-62783844; Fax: 010-62794217; Email: chengq@mail.tsinghua.edu.cn

Correspondence may also be addressed to Chunbo Lou (Email: louchunbo@im.ac.cn) or Bo Yu (Email: yub@im.ac.cn)

[†]The authors wish it to be known that, in their opinion, the first two authors should be regarded as Joint First Authors.

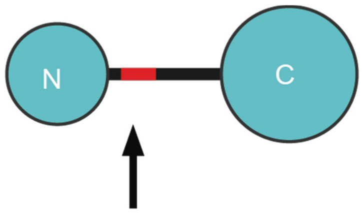

TEV protease cleavage site

...SALGVSDWLLNGTSDSNVRENLYFQGFVGHVEPKGKYQLISMVRAG...

Figure S1. Schematic diagram of the designed CI434-tevS. TEV protease cleavage site (ENLYFQG) is inserted into the linker region between N-terminus and C-terminus domains of CI434 (between the 69th and 70th amino acids). The detailed DNA sequence is listed in Supplementary Table S8**.**

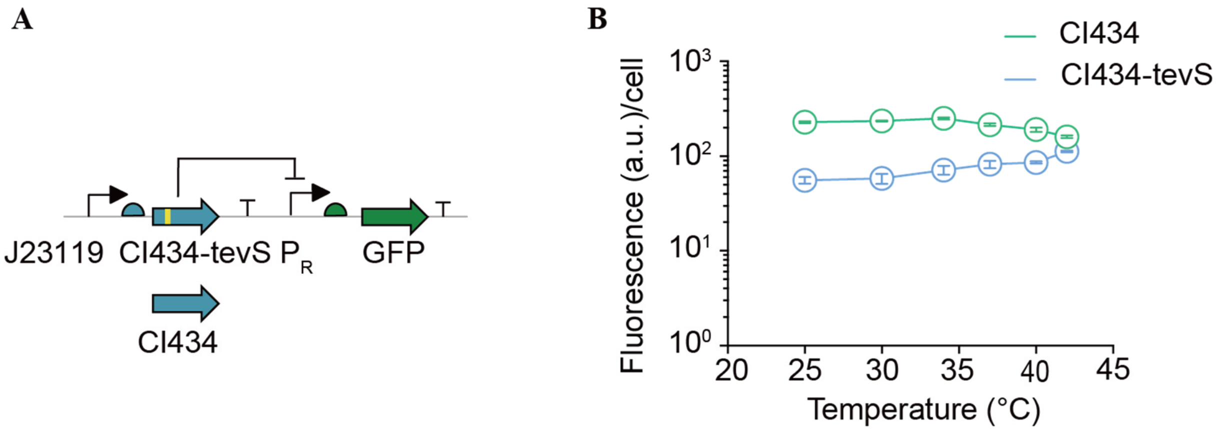

Figure S2. Analysis of the repression of CI434-tevS.

(**A**) Constructs used to test the repression of CI434-tevS. Reporter gene *sf-gfp* is controlled by the cognate promoter (P_R) repressed by the wild-type CI434 or its mutant CI434-tevS. (**B**) The GFP expression repressed by CI434 or CI434-tevS as a function of temperature. The fluorescence of GFP is measured by flow cytometry. All the experimental data were repeated at least three times.

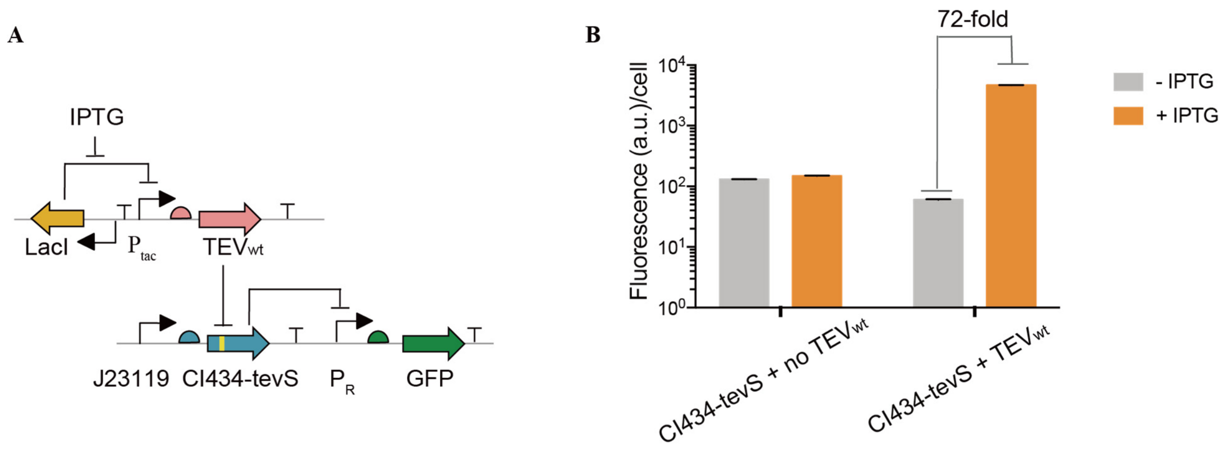

Figure S3. Estimation of the cleavage efficiency of TEV protease on the CI434-tevS repressor.

(**A**) The circuit used to evaluate the cleavage efficiency of TEV protease on the CI434-tevS repressor. (**B**) The GFP expression indicating the cleavage efficiency of TEV protease on the CI434-tevS repressor in both low(-IPTG) and high(+IPTG) TEV expression. The expression of GFP was measured by flow cytometry. All the experimental data were repeated at least three times.

Table S1. Mutations in the selected TF_{ts} and TEV_{ts}

Mutants	Missense Mutations	Synonymous Mutations	Deletion Mutations
TF_{ts}-2	P81Q	/	/
TF_{ts}-10	P81Q, C29S, N194D	E164 (gaa →gag)	/
TF_{ts}-49	F208V	T160 (act → acc)	/
TF_{ts}-50	N36T	Y107 (tat → tac)	/
TEV_{ts}-6	F37S	/	N236 (aat → aa-)<br>C-terminus: NELVYSQ* → KN*
TEV_{ts}-7	P13S, F37S	/	/
TEV_{ts}-11	R80G,156A	/	/
TEV_{ts}-17	N12S, P13S, F37S	/	N236 (aat → aa-)<br>C-terminus: NELVYSQ* → KN*
TEV_{ts}-18	P13S, F37S	/	N236 (aat → aa-)<br>C-terminus: NELVYSQ* → KN*

The PCR templates for TF mutants and TEV mutants are genes of CI434-tevS and TEV_{S219V}, a TEV variant containing S219V mutation. Deletion mutations at N236 (aat → aa-) causes a premature stop codon, which changes the TEV C-terminus from NELVYSQ* to KN* (* refers to the stop codon).

Table S2. Dynamic range of the selected TF_{ts} and TEV_{ts}

Mutants	Dynamic range	s.e.m. (±)	Transition Point (°C)
TF_{ts}-2	4.27	0.21	33
TF_{ts}-10	1.87	0.24	32.5
TF_{ts}-49	4.15	0.65	39
TF_{ts}-50	2.2	0.23	34
TEV_{ts}-6	34.46	5.26	34.5
TEV_{ts}-7	8.34	0.58	39
TEV_{ts}-11	18.75	0.39	33
TEV_{ts}-17	1.7	0.40	38.5
TEV_{ts}-18	2.97	0.27	38.5

Transition point is defined as the temperature at which the fluorescence intensity is reduced to 20% of the maximum fluorescence intensity.

Table S3. Performance of the bioswitch combinations of different TF_{ts} and TEV_{ts}

Bioswitch	Dynamic range	s.e.m. (±)
TF_{ts}-2 & TEV_{ts}-6	121.52	1.62
TF_{ts}-2 & TEV_{ts}-7	269.21	10.95
TF_{ts}-2 & TEV_{ts}-11	211.07	1.72
TF_{ts}-2 & TEV_{ts}-17	65.54	4.65
TF_{ts}-2 & TEV_{ts}-18	349.66	28.71

Dynamic range is defined as the ratio of the maximum and minimum fluorescence intensity over the temperatures ranging from 25°C to 42°C .

Table S4. List of culture media

Culture Medium	Composition	Content
LB	Tryptone	1%(w/v)
	Yeast Extract	0.5%(w/v)
	NaCl	1%(w/v)
SOC	Tryptone	2%
(TaKaRa)	Yeast extract	0.5%
	NaCl	10 mM
	KCl	2.5 mM
	$MgSO_4$	10 mM
	$MgCl_2$	10 mM
	Glucose	20 mM
TB	Tryptone	2%
(Leagene)	Yeast extract	2.4%
	K_2HPO4	72 mM
	KH_2PO4	17 mM
	Glycerol	0.4%
M9	1X M9 salts	
	Glucose	0.4%
	$MgSO_4$	2mM
	$CaCl_2$	0.1mM
	1X amino acid solution	
	1X vitamine solution	
1X M9 salts	$Na_2HPO_4.7H_2O$	6.4%(w/v)
	KH_2PO_4	1.5%(w/v)
	NaCl	0.25%(w/v)
	NH4Cl	0.5%(w/v)

Culture media SOC and TB were purchased from TaKaRa (Tokyo, Japan) and Leagene (Beijing, China) respectively. Tryptone and Yeast Extract were obtained from OXOID (Hampshire, UK). 50X MEM Amino acid and 100X MEM vitamin solution were supplied by Life Technology (MD, USA). Other reagents were purchased from Sinopharm Chemical Reagent Co., Ltd (Beijing, China)

Table S5. Strains used in this study

E. coli strain	Description	Source
TOP10	K-12 *F- mcrA Δ(mrr-hsdRMS-mcrBC) φ80lacZΔM15ΔlacX74 nupG recA1 araD139 Δ(ara-leu)7697 galE15 galK16 rpsL(StrR) endA1 λ-*	Biomed
MG1655	K-12 *F⁻ λ⁻ ilvG⁻ rfb-50 rph-1*	(1)
JM109	K-12 *endA1 glnV44 thi-1 relA1 gyrA96 recA1 mcrB+ Δ(lac-proAB) e14- [F' traD36 proAB+ lacIq lacZΔM15] hsdR17(rK-mK+)*	TaKaRa
BL21	*E. coli* B *F–ompT gal dcm lon hsdSB(rB–mB–) [malB+]K-12(λS)*	TaKaRa
Rosetta(DE3)	*E. coli* B *F-ompThsdSB(r_{B-} m_{B-})galdcmlacY1(DE3)pRARE(argU, argW, ileX, glyT, leuW, proL)(Cam^r)*	Biomed
DH10B	K-12 *F– endA1 deoR+ recA1 galE15 galK16 nupG rpsL Δ(lac)X74 φ80lacZΔM15 araD139 Δ(ara,leu)7697 mcrA Δ(mrr-hsdRMS-mcrBC) StrR λ–*	Biomed
DH5a	K-12 *F– endA1 glnV44 thi-1 recA1 relA1 gyrA96 deoR nupG purB20 φ80dlacZΔM15 Δ(lacZYA-argF)U169, hsdR17(rK–mK+), λ–*	Biomed
JM109SG	*E. coli* JM109 Δsad ΔgabD	(2)
JM109SGΔmreB	*E. coli* JM109 Δsad ΔgabD ΔmreB	(3)
MG1655 P_R- MreB	*E. coli* MG1655ΔmreB: P_R- mreB (Detailed sequences were shown in Supplementary Table S9)	This study
MG1655 P_R-FtsZ	*E. coli* MG1655ΔftsZ: P_R- ftsZ (Detailed sequences were shown in Supplementary Table S9)	This study
MG1655 P_R-FtsZ-pdt#4	*E. coli* MG1655ΔftsZ: P_R- ftsZ-pdt#4 (Detailed sequences were shown in Supplementary Table S9)	This study

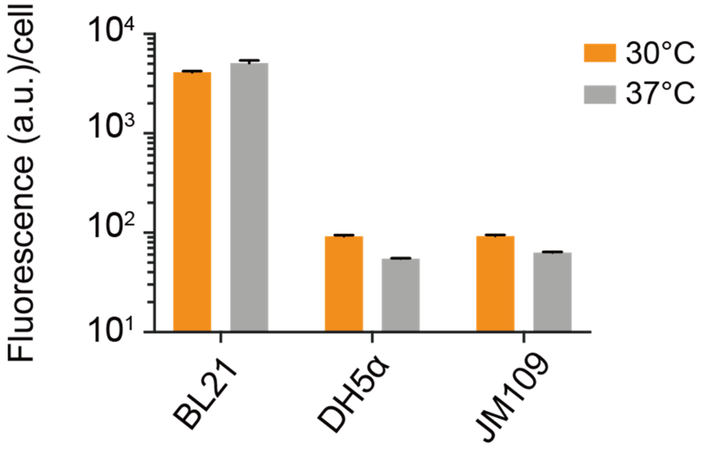

Figure S4. Performance of the unadjusted TEV_{ts}-6&TF_{ts}-2 bioswitch in *E. coli* DH5α, JM109 and BL21, respectively.

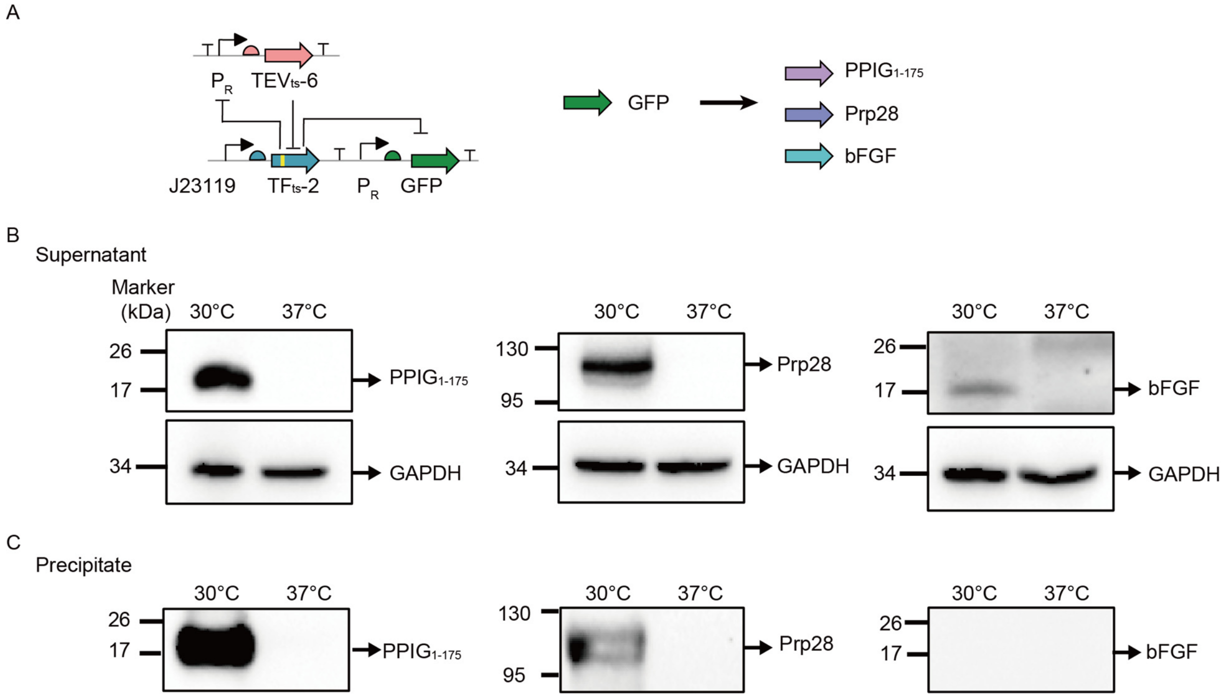

Figure S5. Application of the cold inducible switch for expressing heat-unstable proteins.

(A) Genetic constructs used to express recombinant proteins. Three human recombinant protein genes *PPIG$_{1\text{-}175}$*, *Prp28* and *bFGF* with 6x*His* tag replaced the reporter gene to form three new plasmids: pTFA-TF$_{ts}$-2-PPIG$_{1\text{-}175}$, pTFA-TF$_{ts}$-2-Prp28 and pTFA-TF$_{ts}$-2-bFGF. **(B)** Western blot analysis of the supernatant proteins produced at 30°C and 37°C. *E. coli* strains Rosetta (DE3) containing pPA-TEV$_{ts}$-6 and one new plasmid in (A) were cultured at 30°C and 37°C for 24 h in a LB medium, respectively. Bacteria were harvested by centrifugation at 4°C 14,000 rpm for 10 min. Bacterial pellets were re-suspended in BugBuster (Novagen, WI, USA) protein extraction reagent with protease inhibitor Cocktail Set III (Roche, Switzerland) by pipetting and incubated at room temperature for 20 min. After centrifugation at 4°C 14,000 rpm for 10 min, 120 μL supernatant was treated in boiling water bath for 10 min after being diluted into 30 μL 5XSDS loading buffer. The samples were loaded onto 4-12% SDS-PAGE gel (Invitrogen, CA, USA) for Western blot analysis. GAPDH was used as a loading control for protein normalization. The

loading volume of each sample was regulated based on the first Western blot result of GAPDH. (C) Western blot analysis of the precipitate proteins produced at 30°C and 37°C. 2 mL bacteria were harvested and lysed as described in (B). After centrifugation, the supernatant was discarded. The precipitate was washed by PBS for two times, and then re-suspended in 15 μL PBS containing 8 M urea and heated in in boiling water for 10min. All samples were used for Western blot.

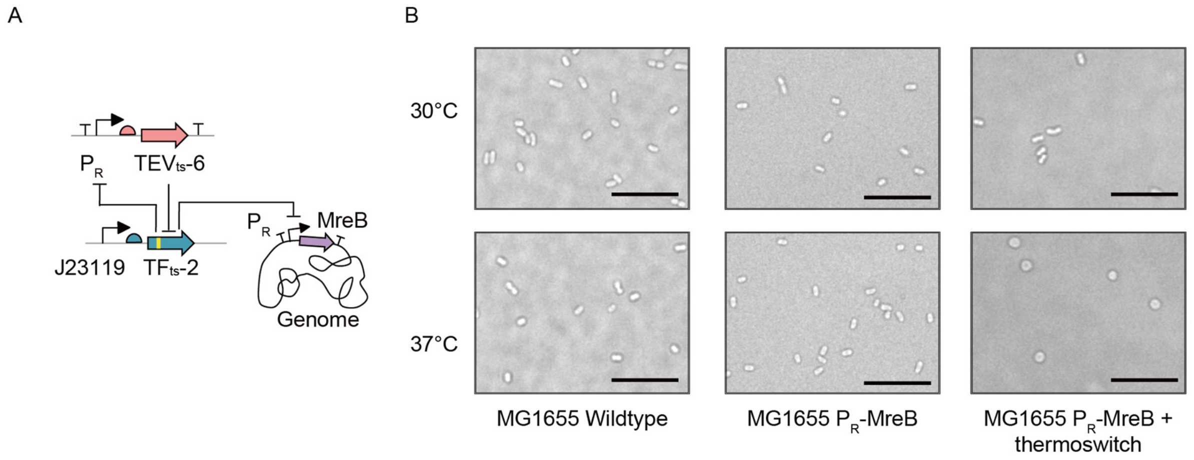

Figure S6. Application of the cold-inducible switch on bacterial morphology by controlling expression of *mre*B gene on the chromosome.

(A) Genetic construct of the cold-inducible switch to regulate endogenous *mreB* on the *E. coli* chromosome. The switchable *mreB* gene expression cassette in plasmid pTFA-TF_{ts}-2-MreB replaces the promoter and RBS of endogenous *mreB*. To avoid interference of the upstream sequence, a terminator was placed before P_R. The detailed sequence is listed in Supplementary Table S9. **(B)** Bacterial morphology for *E. coli* MG1655 with wild-type MreB, constantly expressed MreB or switchable MreB at 30 and 37°C. Microscopy images were taken using an Olympus IX83 (Tokyo, Japan) inverted microscope system, a 100x objective and Olympus CellSens Dimension software (Tokyo, Japan). Scale bar, 10 μm.

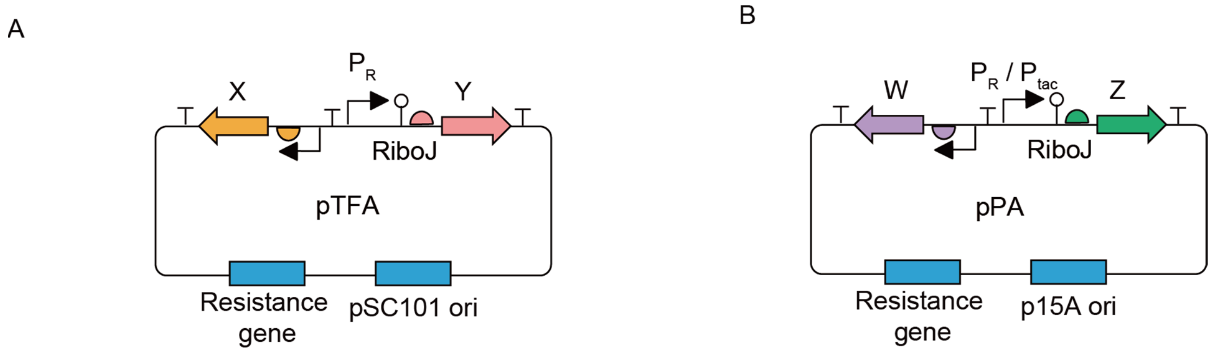

Figure S7. Backbones of plasmid architectures of pTFA and pPA.

The majority of the plasmids used in this study are derived from two basic vectors: pTFA and pPA. The derived plasmids were constructed according to the schemes “pTFA-XY” and “pPA-ZW”, whereby X, Y, Z and W denote the proteins in the corresponding locations. Plasmids used are summarized in Supplementary Table S6. The sequences of the crucial genes, their promoters and ribosome binding sites are listed in Supplementary Table S8.

Table S6. Plasmids used in this study

1.TF associated Plasmids			
Name	X	Y	Source
pTFA-TF_0-sfGFP	CI434	sfGFP	This study
pTFA-TF_{wt}-sfGFP	CI434-tevS	sfGFP	This study
pTFA-TF_{ts}-2-sfGFP	TF_{ts}-2	sfGFP	This study
pTFA-TF_{ts}-10-sfGFP	TF_{ts}-10	sfGFP	This study
pTFA-TF_{ts}-49-sfGFP	TF_{ts}-49	sfGFP	This study
pTFA-TF_{ts}-50-sfGFP	TF_{ts}-50	sfGFP	This study
pTFA-TF_{ts}-2-MreB	TF_{ts}-2	MreB	This study
pTFA-TF_{ts}-2-FtsZ	TF_{ts}-2	FtsZ	This study
pTFA-TF_{ts}-2-FtsZ-pdt#4	TF_{ts}-2	FtsZ-pdt#4	This study
pTFA-TF_{ts}-2-$PPIG_{1\text{-}175}$	TF_{ts}-2	$PPIG_{1\text{-}175}$	This study
pTFA-TF_{ts}-2-Prp28	TF_{ts}-2	Prp28	This study
pTFA-TF_{ts}-2-bFGF	TF_{ts}-2	bFGF	This study
pTFA-TF_{ts}-2-mut3GFP	TF_{ts}-2	mut3GFP	This study
pTFA-TF_{ts}-2-mut3GFP-pdt#4	TF_{ts}-2	mut3GFP-pdt#4	This study
pTFA-mut3GFP	/	mut3GFP	This study

2. Protease associated Plasmids			
Name	Z	W	Source
pPA-TEV_{wt}-LacI	TEV_{S219V} controlled by P_{tac}	LacI	This study
pPA-TEV_{wt}	TEV_{S219V} controlled by P_R	/	This study
pPA-TEV_{ts}-6	TEV_{ts}-6 controlled by P_R	/	This study
pPA-TEV_{ts}-7	TEV_{ts}-7 controlled by P_R	/	This study
pPA-TEV_{ts}-11	TEV_{ts}-11 controlled by P_R	/	This study

pPA-TEV_{ts}-17	TEV_{ts}-17 controlled by P_R	/	This study
pPA-TEV_{ts}-18	TEV_{ts}-18 controlled by P_R	/	This study
pPA-TEV_{ts}-6-TetR-*mf*-Lon	TEV_{ts}-6-TetR controlled by P_R	*mf*-Lon controlled by P_{tetO}	This study

3. Other plasmids

Name	Description	Source
pCas	repA101(Ts) ori, P_{cas}-cas9 P_{araB}-Red lacIq P_{trc}-sgRNA-pMB1	(4)
pTargetF-*pMB1*	pMB1 ori, sgRNA	(4)
pSC101-J23119-sfGFP	pSC101 ori, sfGFP controlled by J23119 promoter	This study

Table S7. Key oligos used in this study

Sequences	5’ → 3’
sgRNA-*ftsZ*	tgattacggcctcaggcgac
sgRNA-*mreB*	cattaagcctttctggactc
RBS of TEV used in *E. coli* TOP10, MG1655, DH10B	aaaaactagagactagtctttaagagaatacca
RBS of TEV used in *E. coli* BL21, Rosetta (DE3)	aaaaactagagacaatcctaagtttgtatacca
RBS of TEV used in *E. coli* JM109 or DH5a	gcacctcaaggaggtggtcat
mf-lon degradation tag pdt#4	gcggcgaacaaaaacgaagaaaacaccaacgaagtgccg acctttatgctgaacgcgggccaggcgaaccacgcccaacct

The sequences of RBS were designed using the RBS calculator V2.0 (5).

Table S8. Sequences of promoters, RBS and representative genes

1. J23119-CI434

ttgacagctagctcagtcctaggtataatgctagcgaataacgtccagtagacgcgtaaatgagtatttcttccagggtaaaagcaaaagaatc
cagcttggacttaaccaggctgaacttgctcaaaaggtggggactacccagcagtctatagagcagctcgaaaacggtaaaactaagcgacc
acgctttttaccagaacttgcgtcagctcttggcgtaagtgttgactggctgctcaatggcacctctgattcgaatgttagatttgttgggcacgttgag
cccaaagggaaatatccattgattagcatggttagagctggttcgtggtgtgaagcttgtgaaccctacgatatcaaggacattgatgaatggtatg
acagtgacgttaacttattaggcaatggattctggctgaaggttgaaggtgattccatgacctcacctgtaggtcaaagcatccctgaaggtcatat
ggtgttagtagatactggacgggagccagtgaatggaagccttgttgtagccaaactgactgacgcgaacgaagcaacattcaagaaactggt
catagatggcggtcagaagtacctgaaaggcctgaatccttcatggcctatgactcctatcaacggaaactgcaagattatcggtgttgtcgtgga
agcgagggtaaaattcgtataa

2. CI434-tevS

atgagtatttcttccagggtaaaagcaaaagaatccagcttggacttaaccaggctgaacttgctcaaaaggtggggactacccagcagtctat
agagcagctcgaaaacggtaaaactaagcgaccacgctttttaccagaacttgcgtcagctcttggcgtaagtgttgactggctgctcaatggca
cctctgattcgaatgttagagagaacctgtacttccagggctttgttgggcacgttgagcccaaagggaaatatccattgattagcatggttagagct
ggttcgtggtgtgaagcttgtgaaccctacgatatcaaggacattgatgaatggtatgacagtgacgttaacttattaggcaatggattctggctgaa
agttgaaggtgattccatgacctcacctgtaggtcaaagcatccctgaaggtcatatggtgttagtagatactggacgggagccagtgaatggaa
gccttgttgtagccaaactgactgacgcgaacgaagcaacattcaagaaactggtcatagatggcggtcagaagtacctgaaaggcctgaac
ccttcatggcctatgactcctatcaacggaaactgcaagattatcggtgttgtcgtggaagcgagggtaaaattcgtataa

3. P_{tac}-TEV_{wt}

tgttgacaattaatcatcggctcgtataatgtgtggaattgtgagcgctcacaattagctgtcaccggatgtgctttccggtctgatgagtccgtgagg
acgaaacagcctctacaaataattttgtttaaaaaaactagagactagtctttaagagaataccaatgggagaaagcttgtttaaggggccgcgt
gattacaacccgatatcgagcaccatttgtcatttgacgaatgaatctgatgggcacacaacatcgttgtatggtattggatttggtcccttcatcatta
caaacaagcacttgtttagaagaaataatggaacactgttggtccaatcactacatggtgtattcaaggtcaagaacaccacgactttgcaacaa
cacctcattgatgggagggacatgataattattcgcatgcctaaggatttcccaccatttcctcaaaagctgaaatttagagagccacaaaggga
agagcgcatatgtcttgtgacaaccaacttccaaactaagagcatgtctagcatggtgtcagacactagttgcacattcccttcatctgatggcatat
tctggaagcattggattcaaaccaaggatgggcagtgtggcagtccattagtatcaactagagatgggttcattgttggtatacactcagcatcga
atttcaccaacacaaacaattatttcacaagcgtgccgaaaaacttcatggaattgttgacaaatcaggaggcgcagcagtgggttagtggttgg
cgattaaatgctgactcagtattgtgggggggccataaagttttcatggtgaaacctgaagagccttttcagccagttaaggaagcgactcaactc
atgaatgaattagtctactcgcaataa

4. P_R-TEV_{ts}-6-TetR

tggatatttacaagaaagtttgttagatttaacgtatgtacaagaaagtttgttgcatcgcgaaccaaaaccagctgtcaccggatgtgctttccggtc
tgatgagtccgtgaggacgaaacagcctctacaaataattttgtttaaaaaaactagagactagtctttaagagaataccaatgggagaaagctt
gtttaaggggccgcgtgattacaacccgatatcgagcaccatttgtcatttgacgaatgaatctgatgggcacacaacatcgttgtatggtattggat
ctggtcccttcatcattacaaacaagcacttgtttagaagaaataatggaacactgttggtccaatcactacatggtgtattcaaggtcaagaacac
cacgactttgcaacaacacctcattgatgggagggacatgataattattcgcatgcctaaggatttcccaccatttcctcaaaagctgaaatttaga
gagccacaaagggaagagcgcatatgtcttgtgacaaccaacttccaaactaagagcatgtctagcatggtgtcagacactagttgcacattcc
cttcatctgatggcatattctggaagcattggattcaaaccaaggatgggcagtgtggcagtccattagtatcaactagagatgggttcattgttggt
atacactcagcatcgaatttcaccaacacaaacaattatttcacaagcgtgccgaaaaacttcatggaattgttgacaaatcaggaggcgcagc
agtgggttagtggttggcgattaaatgctgactcagtattgtgggggggccataaagttttcatggtgaaacctgaagagccttttcagccagttaag
gaagcgactcaactcatgaagaattagtctactcgcaataatactggatccaggaggaaaaaaatgtccagattagataaaagtaaagtgatta
acagcgcattagagctgcttaatgaggtcggaatcgaaggtttaacaacccgtaaactcgcccagaagctaggtgtagagcagcctacattgta
ttggcatgtaaaaaataagcgggctttgctcgacgccttagccattgagatgttagataggcaccatactcacttttgccctttagaaggggaaagc
tggcaagatttttacgtaataacgctaaaagttttagatgtgctttactaagtcatcgcgatggagcaaaagtacatttaggtacacggcctacaga

aaaacagtatgaaactctcgaaaatcaattagccttttatgccaacaaggtttttcactagagaatgcattatatgcactcagcgctgtggggcattt
tactttaggttgcgtattggaagatcaagagcatcaagtcgctaaagaagaaagggaaacacctactactgatagtatgccgccattattacgac
aagctatcgaattatttgatcaccaaggtgcagagccagccttcttattcggccttgaattgatcatatgcggattagaaaaacaacttaaatgtgaa
agtgggtcctaa

5. P_{tetO}-*mf*-Lon

gagtcactaagggctaactaactaattacgtagcaatcaactcactggctcaccttcacgggtgggcctttcttcggcacgggcaaattgctgaat
attccttaccattattatcatgacattaacctataaaaataggcgtatcacgaggcagaatttcagataaaaaaaatccttagctttcgctaaggatg
atttctggaattctctagatttttaattcctaatttttgttgacactctatcgttgatagagttattttaccactccctatcagtgatagagaaaagaattcaa
aagatctaaaactagagactagtctttctcactataccaatgtctaaaaaaatcaaactgccgatcttccagatccgtggttctttcatcgttccggg
tatcaaagaaaacctggaagttggtcgtaaaaacaccctggcttctgttaactacgctatcaaaaactctaacaaccagatgatcgctatcccgc
agatcgacgcttctgttgaaaaaccggagttctctgacctgcacgagttcggtatcctgatcgacttcgaagttatcaaagaatggaaagacaact
ctctgaccatctctaccaacccgatccagcgttgcaaagttatctctttcttcgaaaacgaagaccaggttccgtacgctgaagttgaactgatcga
atctatcaacgacttctctgacgaagaactgaaagaactgatcgaaaaaatctctgacgctatcaaaaccaaggcttctctggttaccaaacaga
tcaaacagctgatctctggtgaatctgacgacctgtctctggctttcgactctatcatgttcaaactggctccgtctaaaatcctgaccaacccggaat
acatcacctctccatctctgaaaacccgttggtctatcatcgaaaaaatcatcttcgctgaagacggtatcatcacccgtaacgctgaatctatcga
cgctgctcgtcagaaaaacgaaatcgaacaggaactgaaccacaaactgaaagaaaaaatggacaaacagcagaaagaatactacctgc
gtgaaaaaatgcgtatcatcaaagacgaactggaaaacctgtacttccagggcgactcttctctggaaaaatacaaagaacgtctggctaaag
aaccgttcccggaagaagttaaacgtaaaatcatggcttctatcaaacgtgttgaagctctccagtctggaaccccggaatggaacaccgaaaa
aaactacatcgactggatgatgtctatcccgtggtgggaagaaaccgaagacctgaccgacctgaaatacgctaaaaaaatcctggacaaac
accactacggtatgaaaaaagttaaagaacgtatcatcgaatacctggctgttaaaaccaaaaccaaatctctgaaagctccgatcatcaccct
ggttggtccgccgggtgttggtaaaacctctctggctaaatctatcgctgaagctgttggtaaaaacttcgttaaagtttctctgggtggtgttaaagac
gaatctgaaatccgtggtcaccgtaaaacctacgttggttctatgccgggtcgtatcatccagaccatgaaacgtgctaaagttaaaaacccgctg
ttcctgctggacgaaatcgacaaaatggcttctgaccaccgtggtgacccggcttctgctatgctggaagttctggacccggaacagaacaaag
agttctctgaccactacatcgaagaaccgtacgacctgtctcaggttatgttcatcgctaccgctaactacccggaagacatcccggaagctctgt
acgaccgtatggaaatcatcaacctgtcttcttacaccgaaatcgaaaaagttaaaatcgctcaggactacctggttccgaaagctatcgaacag
cacgaactgacctctgaagaaatctctttcaccgaaggtgctatcaacgaaatcatcaaatactacacccgtgaagctggtgttcgtcagctgga
acgtcacatcaactctatcatccgtaaatacatcgttaaaaacctgaacggtgaaatggacaaaatcgttatcgacgaaaaacaggttaacgac
ctgctgggtaaacgtatcttcgaccacaccgaaaaacaggaagaatctcagatcggtgttgttaccggtctggcttacacccagttcggtggtgac
atcctgccgatcgaagtttctctgtacccgggtaaaggtaacctgatcctgaccggtaaactgggtgaagttatgaaagaatctgctaccatcgctc
tgacctacgttaaatctaacttcgaaaaattcggtgttgacaaaaaagttttcgaagaaaacgacatccacgttcacgttccggaaggtgctgttcc
gaaagacggtccgtctgctggtatcaccatcaccaccgctctgatctctgctctgtctgacaaaccggtttctaaagaaatcggtatgaccggtga
aatcaccctgcgtggtaacgttctgccgatcggtggtctgcgtgaaaaatctatctctgcttctcgttctggtctgaaaaccatcatcatcccgaaaa
aaaacgaacgtgacctggacgaaatcccggacgaagttaaagctaaactgaaaatcatcccggctgaaaaatacgaagaagttttcgctatc
gttttcaaaaccaaataa

For all the listed DNA sequences in Supplementary Tables S8, the orange, yellow, blue, green sequences are promoters, RiboJ insulators, RBS sequences and terminators, respectively. Other black sequences are coding sequences or linker sequences.

Table S9. Homologous recombination sequences of the target genes

1. Upstream homology arm- P_R-*ftsZ*- downstream homology arm

attaaagttcgccacggttgtgcgctgggttccatcgttggaaaagatgagagcgtggaagtgccgagcgtaggtggtcgtccgccacggagtct
gcaacgtcagacactggcagaggtgatcgagccgcgctataccgagctgctcaacctggtcaacgaagagatattgcagttgcaggaaaagc
ttcgccaacaaggggttaaacatcacctggcggcaggcattgtattaaccggtggcgcagcgcagatcgaaggtcttgcagcctgtgctcagcg
cgtgtttcatacgcaagtgcgtatcggcgcgccgctgaacattaccggtttaacggattatgctcaggagccgtattattcgacggcggtgggattg
cttcactatgggaaagagtcacatcttaacggtgaagctgaagtagaaaaacgtgttacagcatcagttggctcgtggatcaagcgactcaatag
ttggctgcgaaaagagttttaattttttgccacctgacgtctaagaaaaggaatattcagcaatttgcccgtgccgaagaaaggcccacccgtgaa
ggtgagccagtgagttgattgctacgtaattagttagttagcccttagtgactcgaattcgcggccgcttctagagctcggtaccaaattccagaaa
agaggccgcgaaagcggcctttttcgttttggtcctactagatgcctccacaccgctcgtcacatcctggatatttacaagaaagtttgttagatttaa
cgtatgtacaagaaagtttgttgcatcgcgaaccaaaaccagctgtcaccggatgtgctttccggtctgatgagtccgtgaggacgaaacagcct
ctacaaataattttgtttaatactagagaaagaggagaaatactagatgtttgaaccaatggaacttaccaatgacgcggtgattaaagtcatcgg
cgtcggcggcggcggcggtaatgctgttgaacacatggtgcgcgagcgcattgaaggtgttgaattcttcgcggtaaataccgatgcacaagcg
ctgcgtaaaacagcggttggacagacgattcaaatcggtagcggtatcaccaaaggactgggcgctggcgctaatccagaagttggccgcaa
tgcggctgatgaggatcgcgatgcattgcgtgcggcgctggaaggtgcagacatggtctttattgctgcgggtatgggtggtggtaccggtacag
gtgcagcaccagtcgtcgctgaagtggcaaaagatttgggtatcctgaccgttgctgtcgtcactaagcctttcaactttgaaggcaagaagcgta
tggcattcgcggagcaggggatcactgaactgtccaagcatgtggactctctgatcactatcccgaacgacaaactgctgaaagttctgggccg
cggtatctccctgctggatgcgtttggcgcagcgaacgatgtactgaaaggcgctgtgcaaggtatcgctgaactgattactcgtccgggtttgatg
aacgtggactttgcagacgtacgcaccgtaatgtctgagatgggctacgcaatgatgggttctggcgtggcgagcggtgaagaccgtgcggaa
gaagctgctgaaatggctatctcttctccgctgctggaagatatcgacctgtctggcgcgcgcggcgtgctggttaacatcacggcgggcttcgac
ctgcgtctggatgagttcgaaacggtaggtaacaccatccgtgcattgcttccgacaacgcgactgtggttatcggtacttctcttgacccggatat
gaatgacgagctgcgcgtaaccgttgttgcgacaggtatcggcatggacaaacgtcctgaaatcactctggtgaccaataagcaggttcagca
gccagtgatggatcgctaccagcagcatgggatggctccgctgacccaggagcagaagccggttgctaaagtcgtgaatgacaatgcgccgc
aaactgcgaaagagccggattatctggatatcccagcattcctgcgtaagcaagctgattgagaattgactggaatttgggtttcgaggctctttgtg
ctaaactggcccgccgaatgtatagtacacttcggttggataggtaatttggcgagataatacgatgatcaaacaaaggacacttaaacgtatcgt
tcaggcgacgggtgtcggtttacataccggcaagaaagtcaccctgacgttacgccctgcgccggccaacaccggggtcatctatcgtcgcac
cgacttgaatccaccggtagatttcccggccgatgccaaatctgtgcgtgataccatgctctgtacgtgtctggtcaacgagcatgatgtacggattt
caaccgtagagcacctcaatgctgctctcgcgggcttgggcatcgataacattgttatcgaagttaacgcgccggaaatcccgatcatggacgg
cagcgccgctccgtttgtatacctgctgcttgacgccggtatcgacgagttgaactgcgccaaaaaatttgttcgcatcaaag

2. Upstream homology arm-P_R-*ftsZ*-pdt#4- downstream homology arm

attaaagttcgccacggttgtgcgctgggttccatcgttggaaaagatgagagcgtggaagtgccgagcgtaggtggtcgtccgccacggagtct
gcaacgtcagacactggcagaggtgatcgagccgcgctataccgagctgctcaacctggtcaacgaagagatattgcagttgcaggaaaagc
ttcgccaacaaggggttaaacatcacctggcggcaggcattgtattaaccggtggcgcagcgcagatcgaaggtcttgcagcctgtgctcagcg
cgtgtttcatacgcaagtgcgtatcggcgcgccgctgaacattaccggtttaacggattatgctcaggagccgtattattcgacggcggtgggattg
cttcactatgggaaagagtcacatcttaacggtgaagctgaagtagaaaaacgtgttacagcatcagttggctcgtggatcaagcgactcaatag
ttggctgcgaaaagagttttaattttttgccacctgacgtctaagaaaaggaatattcagcaatttgcccgtgccgaagaaaggcccacccgtgaa
ggtgagccagtgagttgattgctacgtaattagttagttagcccttagtgactcgaattcgcggccgcttctagagctcggtaccaaattccagaaa
agaggccgcgaaagcggcctttttcgttttggtcctactagatgcctccacaccgctcgtcacatcctggatatttacaagaaagtttgttagatttaa
cgtatgtacaagaaagtttgttgcatcgcgaaccaaaaccagctgtcaccggatgtgctttccggtctgatgagtccgtgaggacgaaacagcct
ctacaaataattttgtttaatactagagaaagaggagaaatactagatgtttgaaccaatggaacttaccaatgacgcggtgattaaagtcatcgg
cgtcggcggcggcggcggtaatgctgttgaacacatggtgcgcgagcgcattgaaggtgttgaattcttcgcggtaaataccgatgcacaagcg
ctgcgtaaaacagcggttggacagacgattcaaatcggtagcggtatcaccaaaggactgggcgctggcgctaatccagaagttggccgcaa
tgcggctgatgaggatcgcgatgcattgcgtgcggcgctggaaggtgcagacatggtctttattgctgcgggtatgggtggtggtaccggtacag
gtgcagcaccagtcgtcgctgaagtggcaaaagatttgggtatcctgaccgttgctgtcgtcactaagcctttcaactttgaaggcaagaagcgta
tggcattcgcggagcaggggatcactgaactgtccaagcatgtggactctctgatcactatcccgaacgacaaactgctgaaagttctgggccg
cggtatctccctgctggatgcgtttggcgcagcgaacgatgtactgaaaggcgctgtgcaaggtatcgctgaactgattactcgtccgggtttgatg

aacgtggactttgcagacgtacgcaccgtaatgtctgagatgggctacgcaatgatgggttctggcgtggcgagcggtgaagaccgtgcggaa
gaagctgctgaaatggctatctcttctccgctgctggaagatatcgacctgtctggcgcgcgcggcgtgctggttaacatcacggcgggcttcgac
ctgcgtctggatgagttcgaaacggtaggtaacaccatccgtgcatttgcttccgacaacgcgactgtggttatcggtacttctcttgacccggatat
gaatgacgagctgcgcgtaaccgttgttgcgacaggtatcggcatggacaaacgtcctgaaatcactctggtgaccaataagcaggttcagca
gccagtgatggatcgctaccagcagcatgggatggctccgctgacccaggagcagaagccggttgctaaagtcgtgaatgacaatgcgccgc
aaactgcgaaagagccggattatctggatatcccagcattcctgcgtaagcaagctgat**gcggcgaacaaaaacgaagaaaacaccaac**
gaagtgccgacctttatgctgaacgcgggccaggcgaaccacgcccaaccttaagaattgactggaatttgggtttcgaggctctttgtgct
aaactggcccgccgaatgtatagtacacttcggttggataggtaatttggcgagataatacgatgatcaaacaaaggacacttaaacgtatcgttc
aggcgacgggtgtcggtttacataccggcaagaaagtcaccctgacgttacgccctgcgccggccaacaccggggtcatctatcgtcgcaccg
acttgaatccaccggtagatttcccggccgatgccaaatctgtgcgtgataccatgctctgtacgtgtctggtcaacgagcatgatgtacggatttca
accgtagagcacctcaatgctgctctcgcgggcttgggcatcgataacattgttatcgaagttaacgcgccggaaatcccgatcatggacggca
gcgccgctccgtttgtatacctgctgcttgacgccggtatcgacgagttgaactgcgccaaaaaatttgttcgcatcaaag

3. Upstream homology arm-P_R-*mreB* - downstream homology arm

aggccgatgtaggtcaacatatcagtcgtttacaacctgttattcgtttagtgaatgctttaggggtacgggtagccgtcaaccaggctggtttgacg
ctggtaagtaccagttggatcaaagaacttaatgttgagttactcaagctccatccggggctggtcagaaacattgagaagcgaacggagaacc
agctgctggttcaaagcctggtggaagcctgctccgggaccagcacccaggtttacgccaccggcgtgcgttcgcgaagcgagtggcagacc
ctgattcagcgcggtgttacaggcgggcaaggggatttttcgcgtcctcacagccacttgatactaacgtgaaaaaatattcacaaagatactcg
gtttaacctgccgtttaatccgttttcacgtagaataatgcgcgctgcgtctcatgggagtgtgcttgtctgctcgccagattgttgcagcacatatgca
gatgaatgaccttacgcggttgcaaacatgccacctgacgtctaagaaaaggaatattcagcaatttgcccgtgccgaagaaaggcccacccg
tgaaggtgagccagtgagttgattgctacgtaattagttagttagcccttagtgactcgaattcgcggccgcttctagagctcggtaccaaattccag
aaaagaggccgcgaaagcggcctttttcgttttggtcctactagatgcctccacaccgctcgtcacatcctggatatttacaagaaagtttgttagat
ttaacgtatgtacaagaaagtttgttgcatcgcgaaccaaaaccagctgtcaccggatgtgctttccggtctgatgagtccgtgaggacgaaaca
gcctctacaaataattttgtttaatactagagaaagaggagaaatactagatgttgaaaaaatttcgtggcatgttttccaatgacttgtccattgacct
gggtactgcgaataccctcatttatgtaaaaggacaaggcatcgtattgaatgagccttccgtggtggccattcgtcaggatcgtgccggttcaccg
aaaagcgtagctgcagtaggtcatgacgcgaagcagatgctgggccgtacgccgggcaatattgctgccattcgcccaatgaaagacggcgtt
atcgccgacttcttcgtgactgaaaaaatgctccagcacttcatcaaacaagtgcacagcaacagctttatgcgtccaagcccgcgcgttctggttt
gtgtgccggttggcgcgacccaggttgaacgccgcgcaattcgtgaatccgcgcagggcgctggtgcccgtgaagtcttcctgattgaagaacc
gatggctgccgcaattggtgctggcctgccggtttctgaagcgaccggttctatggtggttgatatcggtggtggtaccactgaagtt

For all the listed DNA sequences in Supplementary Tables S9, the orange, yellow, blue, green, magenta sequences are promoters, RiboJ insulators, RBS sequences, terminators and *mf*-Lon degradation tag pdt#4, respectively. Other black sequences are coding sequences or linker sequences.

Table S10. Amino acid sequences of $PPIG_{1-175}$, Prp28 and bFGF.

**1. 6xHis-$PPIG_{1-175}$**
MGSSHHHHHHSSGLVPRGSHMMGIKVQRPRCFFDIAINNQPAGRVVFELFSDVCPKTCENFRCLCTG<br>EKGTGKSTQKPLHYKSCLFHRVVKDFMVQGGDFSEGNGRGGESIYGGFFEDESFAVKHNKEFLLSMA<br>NRGKDTNGSQFFITTKPTPHLDGHHVVFGQVISGQEVVREIENQKTDAASKPFAEVRILSCGELIPK
**2. Prp28-6xHis**
MAGELADKKDRDASPSKEERKRSRTPDRERDRDRDRKSSPSKDRKRHRSRDRRRGGSRSRSRSRS<br>KSAERERRHKERERDKERDRNKKDRDRDKDGHRRDKDRKRSSLSPGRGKDFKSRKDRDSKKDEED<br>EHGDKKPKAQPLSLEELLAKKKAEEEAEAKPKFLSKAEREAEALKRRQQEVEERQRMLEEERKKRKQ<br>FQDLGRKMLEDPQERERRERRERMERETNGNEDEEGRQKIREEKDKSKELHAIKERYLGGIKKRRRT<br>RHLNDRKFVFEWDASEDTSIDYNPLYKERHQVQLLGRGFIAGIDLKQQKREQSRFYGDLMEKRRTLEE<br>KEQEEARLRKLRKKEAKQRWDDRHWSQKKLDEMTDRDWRIFREDYSITTKGGKIPNPIRSWKDSSLP<br>PHILEVIDKCGYKEPTPIQRQAIPIGLQNRDIIGVAETGSGKTAAFLIPLLVWITTLPKIDRIEESDQGPYAII<br>LAPTRELAQQIEEETIKFGKPLGIRTVAVIGGISREDQGFRLRMGCEIVIATPGRLIDVLENRYLVLSRCTY<br>VVLDEADRMIDMGFEPDVQKILEHMPVSNQKPDTDEAEDPEKMLANFESGKHKYRQTVMFTATMPPA<br>VERLARSYLRRPAVVYIGSAGKPHERVEQKVFLMSESEKRKKLLAILEQGFDPPIIIFVNQKKGCDVLAK<br>SLEKMGYNACTLHGGKGQEQREFALSNLKAGAKDILVATDVAGRGIDIQDVSMVVNYDMAKNIEDYIH<br>RIGRTGRAGKSGVAITFLTKEDSAVFYELKQAILESPVSSCPPELANHPDAQHKPGTILTKKRREETIFA<br>EQKLISEEDLLEHHHHHH
**3. bFGF-6xHis**
MAAGSITTLPALPEDGGSGAFPPGHFKDPKRLYCKNGGFFLRIHPDGRVDGVREKSDPHIKLQLQAEE<br>RGVVSIKGVCANRYLAMKEDGRLLASKCVTDECFFFERLESNNYNTYRSRKYTSWYVALKRTGQYKL<br>GSKTGPGQKAILFLPMSAKSEQKLISEEDLLEHHHHHH

Supplementary References

1. Blattner, F.R., Plunkett, G., Bloch, C.A., Perna, N.T., Burland, V., Riley, M., Collado-Vides, J., Glasner, J.D., Rode, C.K., Mayhew, G.F. *et al.* (1997) The complete genome sequence of *Escherichia coli* K-12. *Science*, **277**, 1453-1462.
2. Li, Z.-J., Shi, Z.-Y., Jian, J., Guo, Y.-Y., Wu, Q. and Chen, G. Q. (2010) Production of poly(3-hydroxybutyrate-*co*-4-hydroxybutyrate) from unrelated carbon sources by metabolically engineered *Escherichia coli*. *Metab. Eng.*, **12**, 352-359.
3. Jiang, X.-R., Wang, H., Shen, R. and Chen, G. Q. (2015) Engineering the bacterial shapes for enhanced inclusion bodies accumulation. *Metab. Eng.*, **29**, 227-237.
4. Jiang, Y., Chen, B., Duan, C., Sun, B., Yang, J. and Yang, S. (2015) Multigene editing in the *Escherichia coli* genome via the CRISPR-Cas9 system. *Appl. Environ. Microbiol.*, **81**, 2506-2514.
5. Borujeni, A., Channarasappa, A.S. and Salis, H.M. (2014) Translation rate is controlled by coupled trade-offs between site accessibility, selective RNA unfolding and sliding at upstream standby sites. *Nucleic Acids Res.*, **42**, 2646-2659.
